# LeishIF4E1 Deletion Affects the Promastigote Proteome, Morphology, and Infectivity

**DOI:** 10.1128/mSphere.00625-19

**Published:** 2019-11-13

**Authors:** Nitin Tupperwar, Rohit Shrivastava, Michal Shapira

**Affiliations:** a Department of Life Sciences, Ben-Gurion University of the Negev, Beer Sheva, Israel; University at Buffalo

**Keywords:** CRISPR, *Leishmania*, cap-binding protein, eIF4E

## Abstract

*Leishmania* parasites cycle between sand-fly vectors and mammalian hosts, adapting to changing environmental conditions by driving a stage-specific program of gene expression, which is tightly regulated by translation processes. *Leishmania* encodes six eIF4E orthologs (LeishIF4Es) and five eIF4G candidates, forming different cap-binding complexes with potentially varying functions. Most LeishIF4E paralogs display temperature sensitivity in their cap-binding activity, except for LeishIF4E1, which maintains its cap-binding activity under all conditions. We used the CRISPR-Cas9 system to successfully generate a null mutant of LeishIF4E1 and examine how its elimination affected parasite physiology. Although the LeishIF4E1^–/–^ null mutant was viable, its growth was impaired, in line with a reduction in global translation. As a result of the mutation, the null LeishIF4E1^–/–^ mutant had a defective morphology, as the cells were round and unable to grow a normal flagellum. This was further emphasized when the LeishIF4E1^–/–^ cells failed to develop the promastigote morphology once they shifted from conditions that generate axenic amastigotes (33°C, pH 5.5) back to neutral pH and 25°C, and they maintained their short flagellum and circular structure. Finally, the LeishIF4E1^–/–^ null mutant displayed difficulty in infecting cultured macrophages. The morphological changes and reduced infectivity of the mutant may be related to differences in the proteomic profile of LeishIF4E1^–/–^ cells from that of controls. All defects monitored in the LeishIF4E1^–/–^ null mutant were reversed in the add-back strain, in which expression of LeishIF4E1 was reconstituted, establishing a strong link between the cellular defects and the absence of LeishIF4E1 expression.

**IMPORTANCE** *Leishmania* parasites are the causative agents of a broad spectrum of diseases. The parasites migrate between sand-fly vectors and mammalian hosts, adapting to changing environments by driving a regulated program of gene expression, with translation regulation playing a key role. The leishmanias encode six different paralogs of eIF4E, the cap-binding translation initiation factor. Since these vary in function, expression profile, and assemblage, it is assumed that each is assigned a specific role throughout the life cycle. Using the CRISPR-Cas9 system for *Leishmania*, we generated a null mutant of LeishIF4E1, eliminating both alleles. Although the mutant cells were viable, their morphology was altered and their ability to synthesize the flagellum was impaired. Elimination of LeishIF4E1 affected their protein expression profile and decreased their ability to infect cultured macrophages. Restoring LeishIF4E1 expression restored the affected features. This study highlights the importance of LeishIF4E1 in diverse cellular events during the life cycle of *Leishmania*.

## Abstract (precis)

*Leishmania* parasites are the causative agents of a broad spectrum of diseases. The parasites migrate between sand-fly vectors and mammalian hosts, adapting to changing environments by driving a regulated program of gene expression, with translation regulation playing a key role. The leishmanias encode six different paralogs of eIF4E, the cap-binding translation initiation factor. Since these vary in function, expression profile, and assemblage, it is assumed that each is assigned a specific role throughout the life cycle. Using the CRISPR-Cas9 system for *Leishmania*, we generated a null mutant of LeishIF4E1, eliminating both alleles. Although the mutant cells were viable, their morphology was altered and their ability to synthesize the flagellum was impaired. Elimination of LeishIF4E1 affected their protein expression profile and decreased their ability to infect cultured macrophages. Restoring LeishIF4E1 expression restored the affected features. This study highlights the importance of LeishIF4E1 in diverse cellular events during the life cycle of *Leishmania*.

## INTRODUCTION

Translation in eukaryotes proceeds mostly via cap-dependent mechanisms, whereby the translation initiation complex assembles on the 5′ cap structure of the mRNA. This complex anchors to the mRNA through the translation initiation factor eIF4E, which is a cap-binding protein (1). Most eukaryotes encode several paralogs of eIF4E, with the exception of Saccharomyces cerevisiae, and the detailed functions of the different paralogs are only partially understood. In mammals, eIF4E1 is the canonical translation initiation factor, and eIF4E-2 (also known as eIF4HP) was shown to be a translation repressor; these two paralogs share only 28% homology. eIF4E2 does not bind any eIF4G partner and is assumed to compete with the canonical factor, eIF4E1, for binding to the 5′ cap structure (2, 3). However, it was also reported that under hypoxic conditions, eIF4E2 forms a complex with oxygen-regulated hypoxia-inducible factor 2α (HIF-2α) and the RNA-binding protein RBM4, which is recruited to the RNA hypoxia response element (rHRE) present in specific transcripts. This complex later captures the 5′ cap and targets mRNAs to polysomes for active translation (4). eIF4E3 in eukaryotes binds to the m^7^GTP cap atypically and has been reported to act as a tumor suppressor that competes with the function of canonical eIF4E (5).

Five different cap-binding proteins were identified in Caenorhabditis elegans. These vary in their binding specificities to 7-methylguanosine 5′-triphosphate (m^7^GTP) found on *cis*-spliced transcripts and to 2,2,7-trimethylguanosine (TMG), which is provided by the spliced leader RNA in *trans*-spliced mRNAs. The *Drosophila* genome encodes eight eIF4E isoforms, and the functions of only three isoforms, eIF4E1, eIF4E3, and 4EHP, were determined. The canonical eIF4E1 isoform is ubiquitously expressed, while other isoforms are involved in processes such as embryo development, patterning of oocytes (4EHP), and spermatogenesis in male testes (IF4E3). Translation repression by eIF4E2 (*Drosophila* 4EHP [d4EHP]) is targeted to specific transcripts that are involved in embryo development (6). The overall picture that emerges from studies on 4EHP in various organisms is that modulating the cap-binding activities between the different cap-binding factors is essential for maintaining proper developmental processes, as well as for advancement through the growth cycle of a given cell. The binding affinity of 4EHP to the cap is weaker than that of eIF4E (7), but it is stabilized by an interaction with the help of proteins that specifically bind to 4EHP.

The genome of *Leishmania* encodes six IF4Es (LeishIF4Es), paralogs of the eIF4E cap-binding protein, and five LeishIF4G candidates (8–13). The different LeishIF4Es vary in their cap-binding activities (8) and in their expression profiles throughout the life cycle (14). Each *Leishmania* paralog has orthologs in the genomes of other trypanosomatids, but variations in their specific functions are expected.

LeishIF4E1 binds both the cap-4 and m^7^GTP structures very efficiently. Unlike other paralogs in *Leishmania*, such as LeishIF4E4, that lose their cap-binding activities in axenic amastigotes, LeishIF4E1 continues to actively bind the cap structure under all conditions (14). The use of RNA interference (RNAi)-mediated gene silencing in Trypanosoma brucei revealed that reducing the expression of TbIF4E1 causes a reduction in growth, but when it was combined with silencing of either TbIF4E2 or TbIF4E4, the silencing became lethal. Also, the effect of silencing TbIF4E1 was more profound in the bloodstream forms than in procyclic parasites (15). Similar experiments could not be carried out with *Leishmania* in the absence of a functional RNAi system for these organisms. However, the recent development of the CRISPR-Cas9 system for *Leishmania* (16) now provides a valuable tool to advance our understanding of protein functions by their removal from the genome.

Using the CRISPR-Cas9 system for *Leishmania*, we generated a null mutant of LeishIF4E1 in which both alleles were eliminated. Global translation in the LeishIF4E1^–/–^ mutant cells was impaired, as was cell growth and proliferation. Parasite morphology was changed, and the cells were mostly circular and equipped with a short flagellum. These features were specifically reflected when the amastigote-like cells of the null (LeishIF4E1^–/–^) mutant were switched back to conditions for promastigote growth; the flagellum could not grow, as in promastigotes, and the cells remained circular. Finally, the infectivity of the LeishIF4E1^–/–^ mutant was reduced, emphasizing the potential role of LeishIF4E1 in maintaining parasite virulence.

## RESULTS

### Deletion of LeishIF4E1 by the CRISPR-Cas9 system eliminates its expression completely.

Functional genomic studies of *Leishmania* lag behind those of T. brucei, since RNAi is functional only in the latter. The recent development of the CRISPR-Cas9 system for *Leishmania* improved our ability to carry out such studies with *Leishmania.* To shed new light on the potential role of LeishIF4E1, we attempted to delete its two alleles and examine how this deletion affected various aspects of cell morphology and physiology. The Leishmania mexicana cell line expressing Cas9 and T7 RNA polymerase was first generated by transfection of the pTB007 plasmid (16), followed by selection for hygromycin resistance. LeishIF4E1-specific single guide RNAs (sgRNAs) were then used to target the 5′ and 3′ untranslated regions (UTRs) that flank the LeishIF4E1 open reading frame (ORF) for cleavage and further replacement of the gene with the G418 repair fragment. The LeishIF4E1 deletion cell line was selected in the presence of G418 (200 μg/ml), leading to replacement of both genomic LeishIF4E1 alleles. Selection for G418 resistance was sufficient to eliminate both LeishIF4E1 alleles.

**FIG 1:**
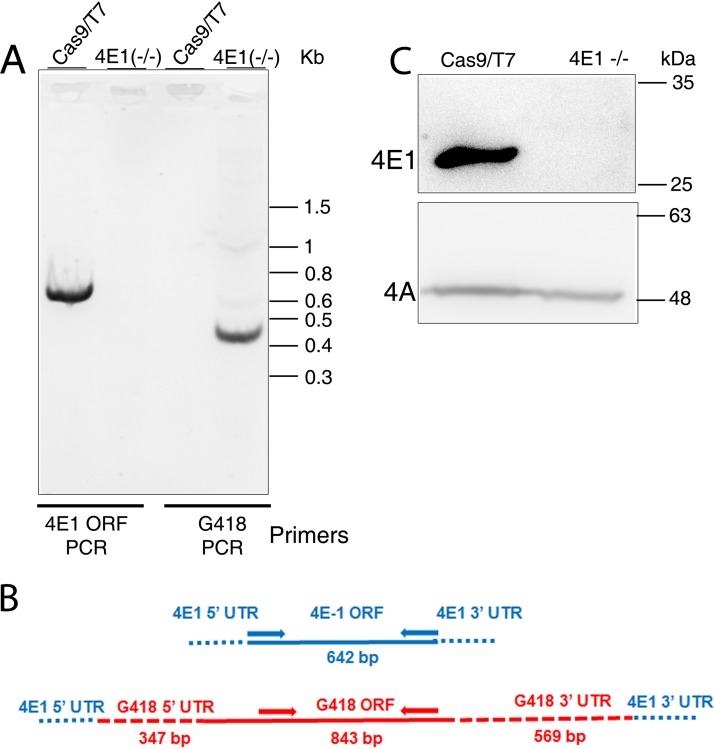
CRISPR-Cas9-mediated deletion of LeishIF4E1. (A) A diagnostic PCR was carried out to confirm the deletion of LeishIF4E1 from the genome of L. mexicana. Genomic DNA extracted from the LeishIF4E1^–/–^ deletion mutant and from the control cell line of L. mexicana expressing Cas9/T7 was used as a template for PCR using primers derived from the LeishIF4E1 ORF and from the G418 resistance gene ORF. (B) Schematic design of the LeishIF4E1 locus and the primers (arrows) used to diagnose the presence or absence of theLeishIF4E1 and G418 resistance genes in the genome of the LeishIF4E1^–/–^ mutant. Primers derived from the LeishIF4E1 ORF are shown in blue. Primers derived from the G418 resistance (G418^r^) gene are shown in red. (C) Western blot using antibodies specific for LeishIF4E1, verifying the absence of LeishIF4E1 in the LeishIF4E1^–/–^ deletion cell line and its presence in the control Cas9/T7 cell line. Cell extracts from the respective cell lines were separated by 12% SDS-PAGE and subjected to Western blot analysis. The interaction with antibodies against LeishIF4A-1 served as a loading control.

The complete elimination of LeishIF4E1 was diagnosed by several PCRs. The reaction that used primers derived from the LeishIF4E1 ORF gave a positive product (642 bp) only when the DNA from the Cas9/T7 cell line served as a template, not with the LeishIF4E1^–/–^ deletion mutant DNA. Another reaction that used primers derived from the G418 resistance gene gave the expected product (450 bp) only when the LeishIF4E1^–/–^ DNA was used as a template for the PCR, not when Cas9/T7 DNA served for this purpose (Fig. 1A and B). These two reactions confirmed the deletion of the LeishIF4E1 gene from the *Leishmania* genome.

The absence of LeishIF4E1 in the mutant cells was also examined by Western blot analysis, using antibodies specific for LeishIF4E1. Figure 1C verifies its absence in the LeishIF4E1^–/–^ deletion mutant, while a clear reaction was observed in Cas9/T7 cell extracts. Antibodies against LeishIF4A were used as a loading control (Fig. 1C). Mass spectrometry (MS) analysis also confirmed the absence of LeishIF4E1, as related peptides were identified only in the lysates of wild-type (WT) cells but were completely excluded from extracts of the LeishIF4E1^–/–^ mutant cell line (see Table S1 in the supplemental material). The combination of the PCR, Western blot, and mass spectrometry analyses validated the successful elimination of LeishIF4E1 from the L. mexicana genome.

10.1128/mSphere.00625-19.9 TABLE S1 Categorized proteins identified in 4E1-KO (knock-out), wild-type, and 4E1 add-back cells. (Sheet 1) Categorized proteome of the identified proteins in LeishIF4E-1KO null mutant cells compared to that of the wild type. The proteomic content of LeishIF4E1KO and WT cells was determined by LC-MS/MS analysis in triplicates. The log_2_ fold differences between the average intensity of each protein identified in 4E1KO and proteins identified in wild-type cells were categorized as downregulated or upregulated. (Sheet 2) Categorized proteome of the identified proteins in 4E1 add-back cells compared to that of LeishIF4E1KO. The proteomic content of LeishIF4E-1 add-back cells and LeishIF4E1KO cells was determined by LC-MS/MS analysis in triplicates. The log_2_ fold difference between the average intensity of each protein identified in LeishIF4E-1 add-back cells and that in LeishIF4E1KO cells was categorized as downregulated or upregulated. Raw mass spectrometric data were analyzed and quantified using the MaxQuant software, and the peptide data were searched against the annotated L. Mexicana proteins listed in TriTrypDB. (Sheet 3) Proteins recovered in the 4E1 add-back which were downregulated in the 4E1^−/−^ mutant line. Upregulated proteins in the 4E1 add-back cells as compared to the downregulated proteins in 4E1^−/−^. Numbers of proteins recovered in 4E1 add-back are represented in the Venn diagram. The number of the downregulated proteins in the 4E1^−/−^ cells is represented in red and the number of recovered proteins in the add-back cells is presented in green. Overlapping proteins are in brown. (Sheet 4) Comparison of the 4E1^−/−^ proteome with published amastigote proteomes. The proteome of 4E1^−/−^ mutant promastigotes was compared with the proteins enriched in the amastigote proteome of virulent *L. amazonensis* PH8 strain, as compared to the less virulent LV79 (27). This paper gives only 261 proteins, which were recovered from infected macrophages and further identified as derived from *Leishmania*. However, there was no comparison with the promastigote proteome so their relative enrichment in amastigotes was not given. The proteins in the 4E1^−/−^ mutant promatigotes were compared to the proteins identified as amastigote specific, following comparison with promastigote proteins. These results were based on 2D gels followed by MS analysis (26). The matched proteins between 4E1^−/−^ mutant (this study) and the proteins that were reported to be enriched in *L. mexicana* amastigotes with the published amastigote proteome are highlighted. Download Table S1, XLSX file, 0.6 MB. Copyright © 2019 Tupperwar et al. 2019 Tupperwar et al. This content is distributed under the terms of the Creative Commons Attribution 4.0 International license.

### Deletion of LeishIF4E1 results in morphological defects in cells grown under promastigote conditions.

**FIG 2:**
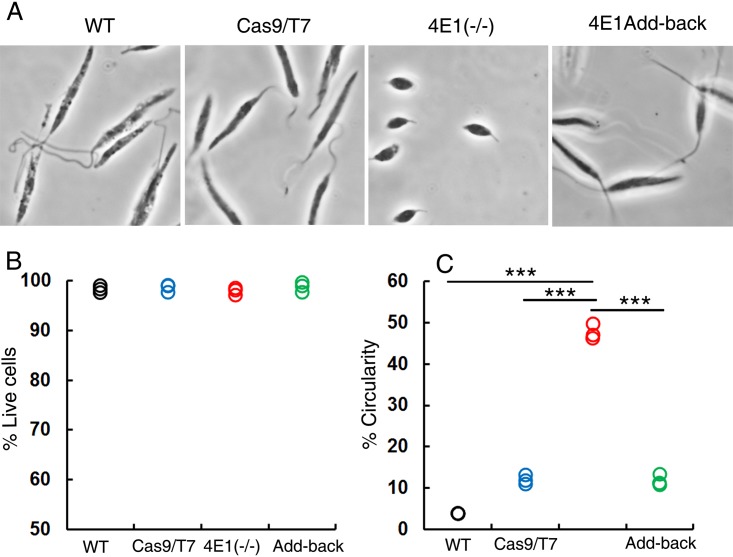
Promastigote morphology is altered in the LeishIF4E1^–/–^ deletion mutant. (A) Promastigotes of LeishIF4E1^–/–^ cells, wild-type cells, Cas9/T7 expressers, and LeishIF4E1 add-back cells from cultures with similar cell counts were fixed with 2% paraformaldehyde and visualized by phase-contrast microscopy at a ×100 magnification. While wild-type, Cas9/T7-expressing, and LeishIF4E1 add-back cells show an elongated cell morphology and a long flagellum, typical of promastigotes, the mutant LeishIF4E1^–/–^ cells are small, round, and equipped with a short flagellum. (B) To verify cell viability, cells were incubated with 20 μg/ml propidium iodide (PI) for 30 min. PI-stained cells were analyzed using the ImageStream X Mark II imaging flow cytometer (Millipore). Twenty thousand cells were analyzed for each sample, and percentages of viable cells were determined. (C) The circularity of single, viable, and focused cells from each of the cell lines was quantified using flow cytometry and is shown as a percentage of the total number of cells measured. Data from three independent experiments are shown.

We monitored the morphology of LeishIF4E1^–/–^ cells grown at 25°C and neutral pH, along with those of wild-type and Cas9/T7 controls and LeishIF4E1 add-back cells, using phase-contrast light microscopy. Several morphological defects were observed in promastigotes of the L. mexicana LeishIF4E1^–/–^ deletion mutant. These mutant cells lost their typical elongated promastigote structure, as reflected by their rounded cell shape and by their shortened flagella (Fig. 2A). These changes were not observed in the control wild-type and Cas9/T7-expressing cells, which were elongated and equipped with long and protruding flagella.

To relate the changes observed in the mutant cell line to the absence of the LeishIF4E1 protein, an add-back cell line in which expression of LeishIF4E1 was recovered by introducing a tagged version of LeishIF4E1 was generated (Fig. S1). LeishIF4E1 expression was recovered by episomal transfection of the deletion mutant cells with pT-Puro-H-LeishIF4E1-SBP-H. Once expression of LeishIF4E1 was recovered, cell morphology and flagellum growth were returned to their normal status, as observed in the wild-type cells (Fig. 2A).

10.1128/mSphere.00625-19.1 FIG S1 LeishIF4E1 protein expression in add-back and wild-type cells. (A) Cell lysates were of L. mexicana LeishIF4E1 add-back cells and were resolved by 10% SDS-PAGE followed by Western blotting with antibodies directed against LeishIF4E-1 (4E1). The Ponceau staining of tubulin on the blots was used as a loading control. LeishIF4E-1 migration in the add-back cells is slower due to the SBP tag, which is absent in the wild-type cells. (B) Densitometry analysis of the change in the steady-state expression of LeishIF4E-1 in add-back cells from that in the wild type, based on three independent repeats. Download FIG S1, PDF file, 0.1 MB. Copyright © 2019 Tupperwar et al. 2019 Tupperwar et al. This content is distributed under the terms of the Creative Commons Attribution 4.0 International license.

Changes in cell shape were also analyzed using flow cytometry. Cell viability was first verified by propidium iodide (PI) staining (Fig. 2B and S2A) of cells that were gated based on being single (nonaggregated) and focused (Fig. S2B). The flow cytometry analysis further served to quantify the relative amounts of circular promastigotes in the different parasite lines relative to that of the cell population that had an elongated shape. The analysis showed that ∼47% of LeishIF4E1^–/–^ mutant cells were circular, while only ∼4% and ∼12% of control wild-type and Cas9/T7 lines, respectively, were circular (Fig. 2C and Fig. S2C). Most importantly, the relative amount of circular cells in the add-back LeishIF4E1–streptavidin-binding peptide (SBP) cells (∼12%) was comparable to that measured for the Cas9/T7 cells (also ∼12%) (Fig. 2C). The reversed phenotype of the LeishIF4E1-SBP add-back cells highlights that the morphological changes in the deletion mutant originated from the absence of LeishIF4E1. These differences were shown to be statistically significant (*P* < 0.001).

10.1128/mSphere.00625-19.2 FIG S2 Flow cytometry for viability, gating of focused single-cell populations, and cell shape quantification. L. mexicana wild-type, control Cas9/T7-expressing, LeishIF4E-1^–/–^ deletion mutant, and LeishIF4E-1 add-back promastigotes were subjected to flow cytometry analysis. (A) Cell viability is represented for focused, singly gated cells for all the different cell lines. (B) Scatterplots representing gated focused single-cell populations for different cell lines. (C) Cell shapes are represented in terms of circularity or elongatedness as scatterplots for the gated cell population. Download FIG S2, PDF file, 0.3 MB. Copyright © 2019 Tupperwar et al. 2019 Tupperwar et al. This content is distributed under the terms of the Creative Commons Attribution 4.0 International license.

### Growth defects and reduced global translation levels in LeishIF4E1^–/–^ cells.

**FIG 3:**
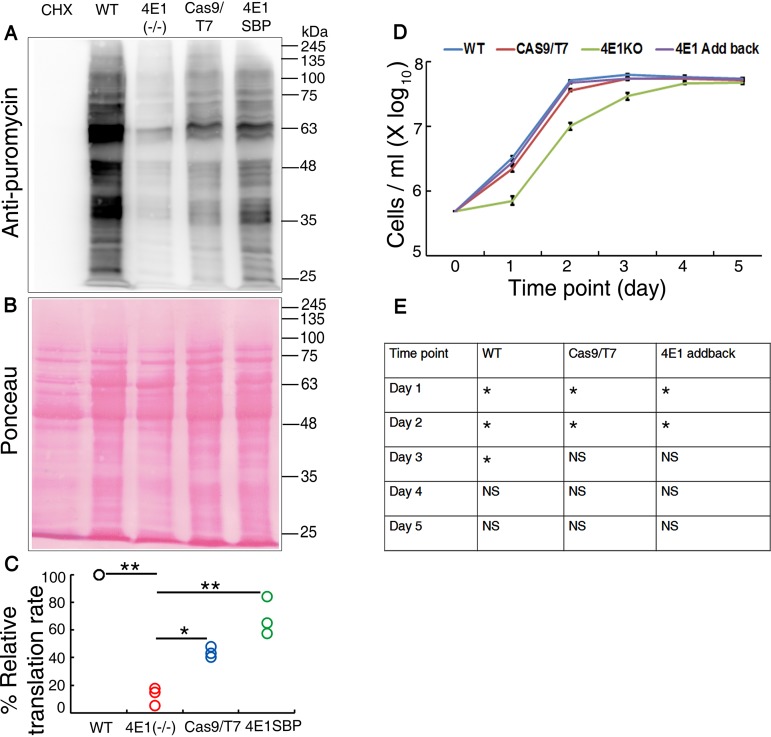
Global translation is reduced in the LeishIF4E1^–/–^ null mutant. (A) LeishIF4E1^–/–^, wild-type, Cas9/T7, and LeishIF4E1-expresing cells were incubated with 1 μg/ml puromycin for 30 min. Cycloheximide (CHX)-treated cells were used a negative control for complete inhibition of translation. Puromycin-treated cells were lysed, resolved by 12% SDS-PAGE, and subjected to Western blot analysis using antibodies against puromycin. (B) Ponceau staining was used to indicate comparable protein loads. (C) Densitometry analysis of puromycin incorporation in the different cell lines, compared to results for wild-type cells (considered 100%). Data from all three independent experiments are represented. (D) The L. mexicana LeishIF4E-1^–/–^mutant, wild-type, and Cas9/T7-expressing control cells, along with LeishIF4E1 add-back promastigotes, were cultured at 25°C in M199 containing essential supplements. Cell counts were monitored daily for 5 consecutive days. The LeishIF4E-1^–/–^ deletion mutant cells are shown in green, wild-type cells in blue, Cas9/T7-expressing cells in brown, and the LeishIF4E-1 add-back cells in yellow. The curves were obtained from three independent assays; error bars are also marked. (E) The statistical analysis demonstrating the significance in the differences between the different cell lines from the growth of the LeishIF4E1^–/–^ mutant was performed by a Kruskal-Wallis test using GraphPad Prism. *, *P <* 0.05. The comparison was done independently for each time point. NS, nonsignificant.

We further investigated the effect of LeishIF4E1 deletion on parasite growth in culture. The LeishIF4E1^–/–^ mutant, along with wild-type and Cas9/T7 controls and the add-back cells, were seeded at an initial concentration of 5 × 10^5^ cells/ml and counted on a daily basis for five consecutive days. The growth curves (Fig. 3D and E) show that the proliferation rate of the LeishIF4E1^–/–^ mutant cells was lower than that of the control wild-type or Cas9/T7-expressing promastigotes, and the LeishIF4E1^–/–^ cells entered their stationary phase of growth at day 4, 2 days after the control cell lines. However, all the cell lines reached similar densities after 5 days in culture. The altered growth rates may be related to the absence of LeishIF4E1, since growth was restored to the level observed in the control lines when the expression of LeishIF4E1 was reconstituted in the add-back cells. The statistical analysis showing the significance of the differences between the cell lines that were tested was performed each day by the Kruskal-Wallis test (Fig. 3E).

We further correlated the lower growth rate of the LeishIF4E1^–/–^ mutant parasites with their global translation rate by using the surface sensing of translation (SUnSET) assay for measuring translation efficiency. This assay monitors puromycin incorporation into *de novo*-synthesized polypeptides, as this drug mimics the structure of tRNA and can be incorporated into growing peptide chains through the ribosomal A site, arresting advancement of the ribosome along the mRNA. The different cell lines were incubated with puromycin (1 μg/ml) for 30 min and then harvested, washed, and analyzed with Western blotting using antipuromycin antibodies. Results shown in Fig. 3A indicate a decrease in the global translation of LeishIF4E1^–/–^ cells compared to that of wild-type and Cas9/T7-expressing control cells, as well as LeishIF4E1-SBP expressers. These were generated by transfection of pX-H-LeishIF4E1-SBP-H into wild-type cells and selected for their G418 resistance. In this translation assay, LeishIF4E1-SBP expressers were used instead of the add-back LeishIF4E1-SBP expressor, since the latter was selected for puromycin resistance, and therefore, puromycin incorporation in the assay might be distorted. The Western blots were further subjected to densitometry analysis, and results were normalized to the protein loads of the gels (Fig. 3B and C). We noticed a significant decrease in the relative translation rate of the LeishIF4E1^–/–^ mutant cells (down to ∼13%) compared to those of the wild type (100%), Cas9/T7-expressing cells (∼44%), and LeishIF4E1-SBP expressers (∼66%). It is important to note that the highest translation was monitored for wild-type cells, whereas control transgenic lines (Cas9/T7 and LeishIF4E1-SBP) showed a lower level of translation. However, the decrease in global translation in the LeishIF4E1^–/–^ deletion mutant was much stronger, supporting the slower growth observed for these cells (Fig. 3D). A cycloheximide-treated sample served as a negative control, with no active puromycin incorporation.

The 2,3-bis [2-methoxy-4-nitro-5-sulfophenyl]-2 H-tetrazolium-5-carboxyanilide inner salt (XTT) assay, which monitors cell metabolism, was also performed to assess the metabolic activities of the mutant and control cells. This assay measures the extracellular reduction of XTT by NADH produced in the mitochondria via *trans*-plasma membrane electron transport and an electron mediator. Figure S3 indicates a reduction of ∼37% in the metabolism of LeishIF4E1^–/–^ mutant cells compared to that of wild-type cells. This difference was shown to be statistically significant (*P* < 0.05).

10.1128/mSphere.00625-19.3 FIG S3 XTT assay for monitoring cellular metabolism. L. mexicana LeishIF4E1^–/–^ and wild-type cells were grown for 2 days in 96-well plates in phenol red-free M199. The calculated optical densities from 450 to 630 nm (OD_450–630_) of the XTT reaction were recorded using an ELISA reader and are presented as means ± SD (*n* = 6). *t* tests (nonparametric) followed by a Wilcoxon matched-pair test were performed to compare the mutant and wild-type cells (*, *P* < 0.05). Download FIG S3, PDF file, 0.09 MB. Copyright © 2019 Tupperwar et al. 2019 Tupperwar et al. This content is distributed under the terms of the Creative Commons Attribution 4.0 International license.

### The LeishIF4E1^–/–^ deletion cells exposed to amastigote growth conditions exhibited a delay in returning to their original form when shifted back to conditions fit for promastigote growth.

**FIG 4:**
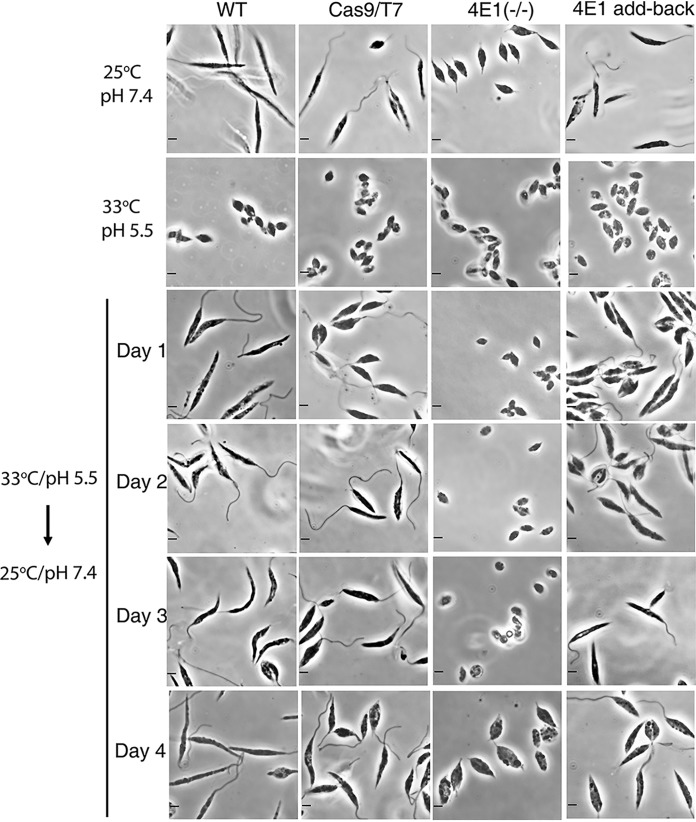
The LeishIF4E1^–/–^ null mutant cells are impaired in their ability to dedifferentiate from amastigote-like cells to promastigotes. Promastigotes of the LeishIF4E1^–/–^ null mutant, wild-type control, Cas9/T7-expressing cells, and LeishIF4E1 add-back cells are shown in the top row. These cells were transferred to conditions known to induce differentiation to axenic amastigotes by their transfer to growth at 33°C and pH 5.5 for 4 days (second row from top). All cell lines were then transferred back to 25°C and pH 7.4 to allow their transformation back to promastigotes. Following this switch, the cells were monitored on a daily basis (rows 3 to 6 from the top). Images were captured at a ×100 magnification with a Zeiss Axiovert 200M microscope equipped with an AxioCam HRm CCD camera. Scale bar, 2 μm.

L. mexicana promastigotes from different cell lines were grown to reach their late log phase of growth and then transferred to conditions that induce differentiation to axenic amastigotes *in vitro* (pH 5.5, 33°C) for 4 days. Cells from the different lines all became small, rounded, and aflagellated, resembling amastigote morphology. The LeishIF4E1^–/–^ cells also became smaller in size. Following this period, the axenic amastigotes were transformed back to conditions fit for promastigote growth, i.e., 25°C and pH 7.4. Under these conditions, all control cell lines transformed back to long, slender, and flagellated promastigotes after 24 h, except for the LeishIF4E1^–/–^ cells, including the wild-type and Cas9/T7 cells. LeishIF4E1^–/–^ cells failed to return to their original size and form for 3 days. Only after 4 days did the cells become larger, although still rounded, and grow a short flagellum, as observed in their original morphology (Fig. 4 and see Fig. S4 for a broad-field view). Reconstituting expression of LeishIF4E1 in the null LeishIF4E1^–/–^ mutant cells by episomal transfection of a vector expressing the tagged LeishIF4E1-SBP expressor restored the ability of these add-back cells to transform back to promastigotes, like wild-type and Cas9/T7 control cells (Fig. 4, right column).

10.1128/mSphere.00625-19.4 FIG S4 The LeishIF4E-1^–/–^ null mutant cells are impaired in their ability to dedifferentiate from amastigote-like cells to promastigotes (broad field). The morphologies of LeishIF4E-1^–/–^ null mutant, control wild-type, Cas9/T7-expressing, and LeishIF4E-1 add-back cells were monitored. Promastigotes of all four cell lines are shown in the top row. These cells were transferred to conditions known to induce differentiation into axenic amastigote-like cells (33°C and pH5.5, with mild shaking) after 4 days (second row from top). All cell lines were then allowed to dedifferentiate back to promastigotes by shifting them to growth at 25°C and pH 7.4. Cell morphologies were monitored on a daily basis (rows 3 to 6 from the top). Images were captured at a ×100 magnification with a Zeiss Axiovert 200M microscope equipped with AxioCam HRm CCD camera. The scale bar marks 5 μm. Download FIG S4, PDF file, 0.2 MB. Copyright © 2019 Tupperwar et al. 2019 Tupperwar et al. This content is distributed under the terms of the Creative Commons Attribution 4.0 International license.

**FIG 5:**
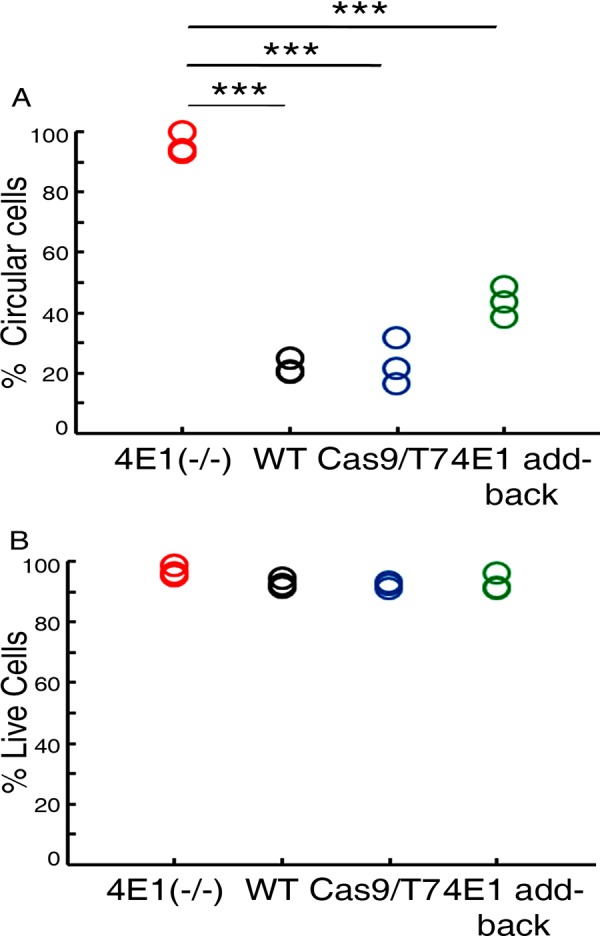
Quantification of cells transformed back to promastigotes. (A) Promastigotes of the LeishIF4E1^–/–^ null mutant, wild-type control, and Cas9/T7-expressing cells, along with LeishIF4E1 add-back cells, were exposed to conditions that induce differentiation into axenic amastigote-like cells (33°C and pH 5.5) for 4 days. Following this period, the cells were transferred back to conditions typical for the growth of promastigotes (25°C and pH 7.4) for 2 days. (B) Cell viability was monitored by the ImageStream X Mark II imaging flow cytometer (Millipore) following incubation of the cells in 20 μg/ml PI for 30 min. Cell morphology was also analyzed in parallel, using the IDEA software. Twenty thousand cells were analyzed for each sample. Data from all three independent experiments are represented.

Further, we quantified the proportion of circular axenic amastigotes in each of the populations, using the IDEAS software that analyzes data obtained by an imaging flow cytometer. Since it was difficult to obtain a population of well-segregated amastigotes due to their tendency to aggregate, we flushed the cells with a 24-gauge needle before flow cytometry analysis and identified the area of the cell population that was nonaggregated, focused, and circular (Fig. 5A), as shown by the imaging flow cytometer image (left panel of Fig. S5A, numbers 1 to 3). Using this approach, we quantified the number of single, rounded, and focused cells in the gated area. This analysis showed that even 2 days after the cells were switched to promastigote growth conditions, LeishIF4E1^–/–^ deletion cells, unlike the wild-type and Cas9/T7 control cells, maintained their small aflagellated and round axenic amastigote-like morphology and failed to transform back to their original form, which featured a short flagellum and larger cells. Figure 5A shows that the majority of the wild-type and Cas9/T7 cells had already converted to flagellated and elongated promastigotes after 1 day (Fig. 4), as quantified after 2 days (Fig. 5A and Fig. S5A), leaving only ∼22% (wild type) and 23% (Cas9/T7) nonflagellated rounded cells, compared to percentages for the LeishIF4E1^–/–^ cells, which retained a maximum proportion of rounded and nonflagellated cells (100%). The recovery of LeishIF4E1 expression in the add-back parasites increased the percentage of cells that adapted the promastigote-like morphology, leaving only 43% of the cell population as nonflagellated and rounded cells. Figure 5A and Fig. S5A indicate that the LeishIF4E1 add-back cells improved their ability to adapt the promastigote-like morphology, although they did so only partially, following their transition to conditions typical of promastigote growth. All the groups show a significant difference in their morphologies from that of the LeishIF4E1^–/–^ mutant cells. The viability of the gated population from all the groups was not affected, as indicated by the negative uptake of PI into the cells (Fig. 5B and Fig. S5B).

10.1128/mSphere.00625-19.5 FIG S5 Flow cytometry analysis of the viability and gating of a single-focus amastigote population. (A) The scatterplot in red represents gating of the focused single-cell amastigote populations in the different cell lines. Numbers 1 to 4 represent the populations of single rounded amastigotes (1), cell aggregates (2), the population of elongated promastigote-like cells (3), and cell debris (4). (B) Cell viability is shown for the gated, focused, singly gated amastigotes in the different cell lines. Download FIG S5, PDF file, 0.2 MB. Copyright © 2019 Tupperwar et al. 2019 Tupperwar et al. This content is distributed under the terms of the Creative Commons Attribution 4.0 International license.

### LeishIF4E1^–/–^ null mutant cells are impaired in their ability to infect macrophages.

**FIG 6:**
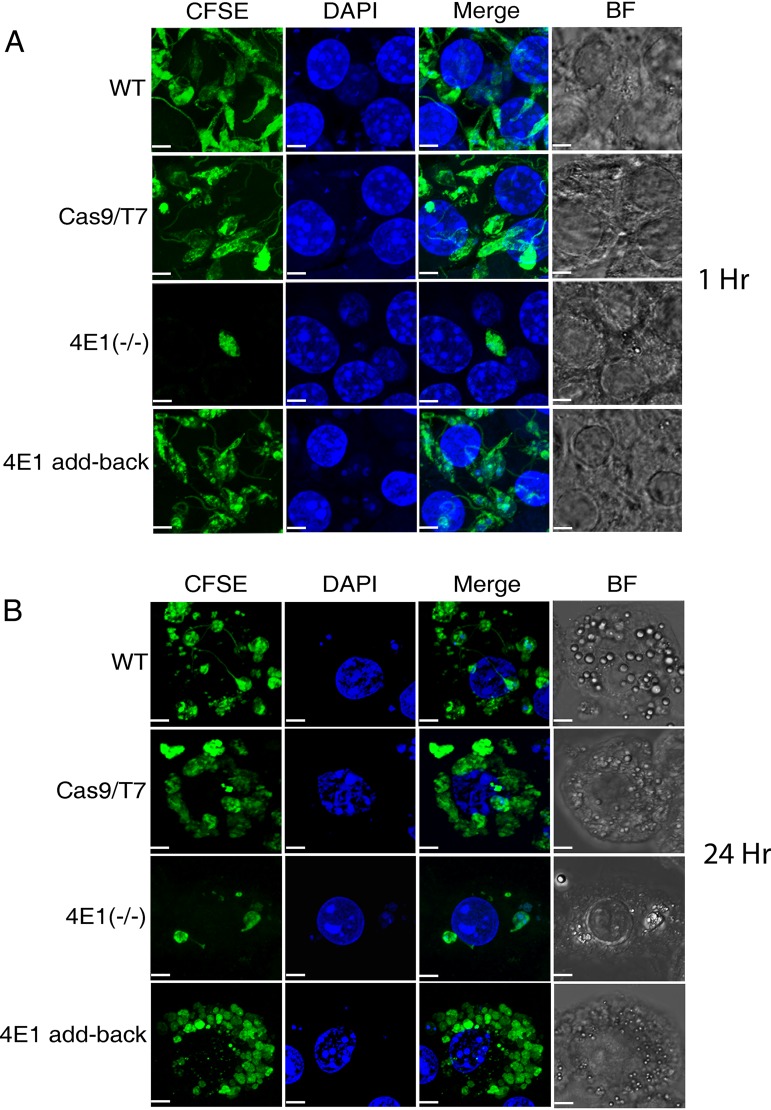
The LeishIF4E1^–/–^ null mutant cells are impaired in their ability to infect cultured macrophages. Stationary-phase L. mexicana LeishIF4E1^–/–^ null mutant, wild-type, and Cas9/T7-expressing cells, along with add-back cells, were prestained with CFSE and further used to infect RAW 264.7 macrophages at a ratio of 10:1 for 1 h. The cells were then washed to remove excess parasites, and the macrophages were cultured for 1 h (A) or 24 h (B) at 37°C. Macrophage nuclei were stained with DAPI, and the infected macrophage slides were processed for confocal microscopy, showing a Z-projection produced by Image J software. Fields containing 200 cells were further evaluated to quantify the infection. The scale bar represents 5 μm.

Given the altered morphology of the LeishIF4E1^–/–^ null mutant cells, their lack of a normal flagellum, their slow growth, and their reduced global translation, we investigated their ability to infect cultured murine macrophage cells using the RAW 267.4 line. *Leishmania* parasites were first prestained with carboxyfluorescein succinimidyl ester (CFSE) and then allowed to infect the macrophages for 1 h at a multiplicity of infection of 10 parasites per macrophage at 37°C. The macrophages were then washed to remove free parasites, and the cells were fixed and processed for confocal analysis. Nuclei were stained with DAPI (4′,6-diamidino-2-phenylindole). The infected macrophage cultures were visualized either immediately (within 1 h) or after a subsequent incubation of the infected macrophages 24 h following infection. This allowed us to monitor the entry of the parasites and further follow their ability to maintain themselves within the infected macrophages. The infectivity of the LeishIF4E1^–/–^ null mutant cells was compared to those of wild-type cells and control Cas9/T7-expressing cells, along with that of Leish4E-1 add-back cell lines. The infectivity index was measured by counting the number of infected macrophages in different fields that added up to 200 macrophages. The results shown in Fig. 6A and B and Fig. S6A and B (for a broad-field view) indicate that the infectivity of the null LeishIF4E1^–/–^ mutant was already impaired at 1 h postinfection and more so after 24 h, compared to those of the wild type and Cas9/T7 expressers and to the add-back controls, all of which showed that between 91 and 94% of the cells were infected. At both time points, the infection of the LeishIF4E1^–/–^ mutant was significantly reduced compared to that of control cells, i.e., to 48% after 1 h and to 66% after 24 h (Fig. S7A). Once the expression of LeishIF4E1 was recovered in the add-back cells, the ability of the parasites to enter into the macrophages was recovered completely by reconstitution of LeishIF4E1 expression.

10.1128/mSphere.00625-19.6 FIG S6 The LeishIF4E-1^–/–^ null mutant cells are impaired in their ability to infect cultured macrophages (broad field). Stationary-phase L. mexicana LeishIF4E-1^–/–^ null mutant, wild-type, and Cas9/T7-expressing cells, along with add-back cells, were prestained with the CFSE dye and further used to infect RAW 264.7 macrophages at a ratio of 10:1 for 1 h. The cells were then washed to remove excess parasites, and the macrophages were cultured for 1 h (A) or 24 h (B) postinfection at 37°C. Macrophage nuclei were stained with DAPI, and the infected macrophages were processed for confocal microscopy. A representative section of Z-projections (maximum intensity) produced by Image J software is presented in the figure. Fields containing 200 cells were further evaluated to quantify the infection. Download FIG S6, PDF file, 0.3 MB. Copyright © 2019 Tupperwar et al. 2019 Tupperwar et al. This content is distributed under the terms of the Creative Commons Attribution 4.0 International license.

10.1128/mSphere.00625-19.7 FIG S7 Statistical analysis of the LeishIF4E-1^–/–^ mutant infectivity compared to those of controls. The parasite infectivity of cultured RAW 264.7 macrophages was estimated *in vitro*, using the Image J software. (A) The percentage of infected macrophages was determined by counting a total of 200 macrophages from three independent experiments. (B) The average number of parasites per infected cell is shown. The Kruskal-Wallis test in GraphPad Prism was performed for the statistical analysis of the percentage of infected cells and for calculation of the average number of parasites per cell, along with standard deviation values. The percentage of infected macrophages and the average number of parasites per macrophage in the LeishIF4E-1^–/–^ deletion mutant were compared with values for each of the control lines: the wild type, Cas9/T7-expressing cells, and LeishIF4E-1 add-back cells. A *P* value of <0.001 is represented by ***, while a *P* value of <0.01 is represented by **. The statistical differences between the control lines were nonsignificant. The data are shown for 1-h and 24-h macrophage infections in separate panels. Download FIG S7, PDF file, 0.3 MB. Copyright © 2019 Tupperwar et al. 2019 Tupperwar et al. This content is distributed under the terms of the Creative Commons Attribution 4.0 International license.

A pronounced inhibitory effect of LeishIF4E1 deletion was observed in the reduced number of parasites per infected macrophage (Fig. 6A and B). While wild-type and Cas9/T7 control cells showed 2.6 parasites per infected macrophage after 1 h of infection, LeishIF4E1^–/–^ cells showed only 1.16 parasites per infected macrophage at that time point. However, LeishIF4E1 add-back cells recovered their ability to infect macrophages, with 2.7 parasites per infected macrophage. The effect of LeishIF4E1 deletion on reducing the number of parasites per infected cell was observed also at 24 h postinfection. Wild-type and Cas9/T7 control cells showed 7.01 and 7.08 parasites per infected macrophage, respectively, and LeishIF4E1^–/–^ cells showed only 2.62 parasites per infected macrophage. LeishIF4E1 add-back cells recovered their ability to infect macrophages, with 6.82 parasites per infected macrophage. The differences in the infectivities of the LeishIF4E1^–/–^ mutant cells as reflected by both the reduced number of infected macrophages and the reduced number of parasites per infected macrophage were significant compared to those after infection with wild-type, Cas9/T7, or LeishIF4E1 add-back cells (Fig. S7A and B).

### Proteomic analysis of the LeishIF4E1^–/–^ mutant cells shows a reduction in cytoskeletal components and flagellar-rod proteins.

**FIG 7:**
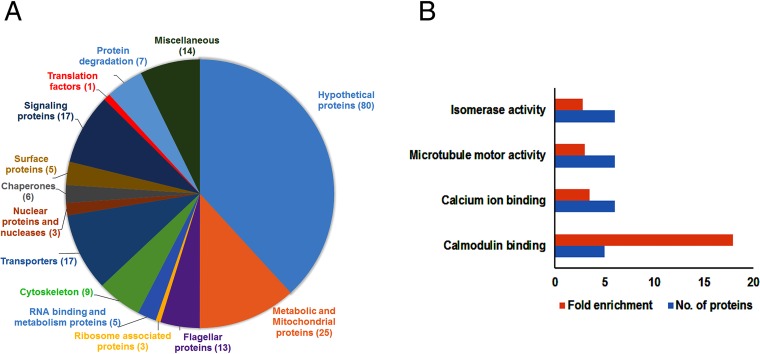
Categorized proteome of the downregulated proteins (compared to the proteome of the WT) in LeishIF4E1^–/–^ null mutant cells. The proteomic contents of LeishIF4E1^–/–^ and WT cells were determined by liquid chromatography-tandem mass spectrometry (LC-MS/MS) analysis in triplicate. Raw mass spectrometric data were analyzed and quantified using the MaxQuant software, and the peptide data were searched against the annotated L. mexicana proteins listed in TriTrypDB. The summed intensities of the peptides that served to identify the individual proteins were used to quantify changes in the proteomic contents of specific proteins. Statistical analysis was done using the Perseus software. Proteins whose expression was reduced in the LeishIF4E1^–/–^ null mutant by 3-fold compared to their expression in WT cell extracts (*P* < 0.05) are shown. (A) Proteins in the LeishIF4E1^–/–^ mutant that were downregulated (>3-fold) compared to their expression in WT extracts were clustered manually into functional categories. The pie chart represents the summed intensities of downregulated or deleted protein categories in the LeishIF4E1^–/–^ null mutant. Numbers in parentheses indicate the number of proteins in each category. (B) Enriched proteins were classified by the GO enrichment tool in TriTrypDB, based on molecular function. The threshold for the calculated enrichment of proteins based on their GO terms was set for 2.5-fold (*P* < 0.05). This threshold eliminated most of the general groups that represented parental GO terms. GO terms for which only a single protein was annotated were filtered out as well. In some cases, GO terms that were included in other functional terms are not shown, leaving only the representative GO term.

To investigate potential differences in the proteomic profiles that resulted from deletion of LeishIF4E1, we carried out mass spectrometry analysis of the total cell extracts from LeishIF4E1^–/–^ mutant cells and compared them to those of wild-type and add-back cells. Day 2 logarithmic cells were used for this analysis. This analysis was performed with three independent samples that were analyzed in parallel and in the same run. The resulting peptides were identified by their comparison to the genome of L. mexicana in TriTrypDB, and these were further quantified by the MaxQuant software. The proteomic content of the LeishIF4E1^–/–^ cells was compared to that of control wild-type cells to identify proteins that were relatively decreased, using a threshold of at least 3-fold (*P* < 0.05). The statistical analysis was carried out by the Perseus software platform (17) (Table S1). Based on the results obtained, we categorized the proteins in the LeishIF4E1^–/–^ cells that were downregulated by at least 3-fold compared to their expression in wild-type cells into different groups, based on their known functions. Figure 7A and Table S1 describe the manually categorized groups of 191 downregulated proteins and highlight a strong decrease in the expression of the core proteins of the paraflagellar rod as well as of proteins that relate to the flagellum itself and cytoskeleton. A decrease in expression was also noticed in proteins that were reported to be associated with the flagellum and flagellar rod, such as Ca^2+^-sensing and calmodulin-binding proteins (18). The relative abundances of surface proteins, such as GP63 and GP46/PSA (PSA stands for promastigote surface antigen), were also reduced; both are known to be involved in parasite virulence (19–22). In addition, we measured decreased expression of proteins involved in metabolism, including mitochondrial proteins, correlating with the reduced metabolism demonstrated by the XTT assay. As expected, RNA-binding proteins and proteins involved in RNA metabolism also decreased in the total proteome of LeishIF4E1^–/–^ mutant cells.

The downregulated proteins were also subjected to gene ontology (GO) enrichment analysis through the TryTripDB platform, based on their molecular functions. Figure 7B and Table S2 highlight the major categories of the downregulated protein groups, each containing at least 5 proteins. In line with the manually categorized proteins, the GO enrichment analysis also found that the downregulated proteins relate to the microtubule motor activity. Other groups that were downregulated in the GO enrichment analysis included the calmodulin- and calcium-binding proteins. The same group also included PFR1 (LmxM.08_29.1760) and PFR2 (LmxM.16.1430) (Table S2), known to be associated with the paraflagellar rod. As with the manual categorization, the GO enrichment analysis highlighted a decrease in metabolic pathways, such as isomerase activity. We also noticed the upregulation of 141 proteins in LeishIF4E1^–/–^ cells compared to their expression in wild-type cells. These included proteins involved in gluconeogenesis, a process that increases in amastigotes (23), and several enzymes involved in fatty acid metabolism, although their number was limited. These may indicate some resemblance with amastigote functions, although very limited. It was interesting to note that kinesins were upregulated in the LeishIF4E1^–/–^ mutant but that dyneins were downregulated (Table S1). These two groups of proteins represent opposite movements along microtubules (24, 25) and may be related to changes in cell division.

10.1128/mSphere.00625-19.10 TABLE S2 Proteomic content of downregulated proteins in 4E1 KO cells versus WT cells and in 4E1 add-back cells versus 4E1 KO cells, classified by GO term enrichment. The proteomic content was assessed by LC-MS/MS as described in Materials and Methods. The differentially enriched proteins given in Table S1 were analyzed by the GO term enrichment tool, based on their molecular functions. All GO terms were enriched by at least 2.5-fold compared to the gene sets encoded in the genome (*P* < 0.05). Download Table S2, XLSX file, 0.01 MB. Copyright © 2019 Tupperwar et al. 2019 Tupperwar et al. This content is distributed under the terms of the Creative Commons Attribution 4.0 International license.

To further strengthen the association between the downregulated protein groups and the absence of LeishIF4E1, we show that recovery of LeishIF4E1 expression in the add-back cells led to the upregulation of 155 of the 204 proteins that were downregulated in the mutant LeishIF4E1^–/–^ cells (Table S1). This is shown in the Venn diagram derived from the manually categorized proteins. The GO enrichment groups of proteins that were upregulated in the add-back cell line is also shown (Fig. S8 and Tables S1 and S2). The upregulated proteins included the paraflagellar-rod proteins, calmodulin-binding, and Ca^2+^-sensing proteins PFR1 and PFR2, along with cytoskeletal proteins (Tables S1 and S2). The proteome of LeishIF4E1^–/–^ cells was also compared with the L. mexicana and Leishmania amazonensis proteins that were upregulated in L. mexicana amastigotes (26) or in a virulent strain of L. amazonensis (27). However, we observed an overlap of only 4 proteins that were upregulated in the LeishIF4E1^–/–^ mutant with L. mexicana amastigote-specific proteins (Tables S1). These were proteins that are known to be involved in fatty acid metabolism.

10.1128/mSphere.00625-19.8 FIG S8 Categorized proteome of the up-regulated proteins in Leish4E1 add-back cells (compared to their expression in LeishIF4E-1^–/–^ cells). The proteomic contents of Leish4E1 add-back and LeishIF4E-1^–/–^ cells were determined by LC-MS/MS analysis in triplicates. MaxQuant software using TriTrypDB database annotations were used to identify the L. mexicana proteins. Proteins that were upregulated in Leish4E1 add-back cells with a 3-fold difference and with a *P* of <0.05 compared to protein expression in LeishIF4E-1^–/–^ cells were determined using the Perseus statistical tool, and differences were considered significant. (A) Upregulated or deleted proteins in Leish4E1 add-back cells compared to those in LeishIF4E-1^–/–^ cells were clustered manually into different functional categories. The pie chart represents the relative distribution of upregulated proteins in Leish4E1 add-back cells into different categories, based on the total intensities of the peptides that were used to identify the individual proteins. The numbers in parentheses in the pie chart represent the number of proteins in each category. (B) Gene ontology enrichment by molecular function. Upregulated proteins in Leish4E1 add-back cells were subjected to the gene ontology enrichment tool (available at https://tritrypdb.org/tritrypdb/) with molecular functions. The resulting GO terms were enriched by at least 2.5-fold (*P* < 0.05). Download FIG S8, PDF file, 0.2 MB. Copyright © 2019 Tupperwar et al. 2019 Tupperwar et al. This content is distributed under the terms of the Creative Commons Attribution 4.0 International license.

## DISCUSSION

The six *Leishmania* cap-binding protein paralogs vary from each other in their sequences, affinities to the mRNA cap structures, and binding partners. These variations suggest that each LeishIF4E paralog is responsible for different functions, although overlaps are also expected (15, 28). This study aimed to investigate the role of LeishIF4E1 in *Leishmania* by testing how its deletion affected the proteomic profile of the deletion mutant and the subsequent effect on broad aspects of parasite morphology, metabolism, growth, and virulence.

Previous studies on the different cap-binding protein paralogs in *Leishmania* showed that LeishIF4E4 anchors a conventional cap-binding complex that contains LeishIF4G3 and LeishIF4A1, but it was shown to be sensitive to temperature stress (14). LeishIF4E3, another eIF4E isoform, has a relatively weak cap-binding activity and was shown to be involved in the formation of storage granules during nutritional stress; it too loses its activity at elevated temperatures (29, 30). Among the tested paralogs, the only LeishIF4E isoform that maintains its cap-binding activity at elevated temperatures and under conditions that generate axenic amastigotes is LeishIF4E1 (14). However, we could not demonstrate an interaction between LeishIF4E1 and any LeishIF4G paralog (14), which is rather unusual among canonical eIF4Es in higher eukaryotes (1). Furthermore, we previously reported on a 4E-interacting protein (Leish4E-IP) that binds exclusively to LeishIF4E1 (14) and interferes with its cap-binding activity (31), suggesting that the activity of cap-binding proteins in *Leishmania* is tightly regulated. However, despite the accumulated body of structural and molecular information, the function of LeishIF4E1 is still not fully understood.

In the absence of an RNAi system in *Leishmania*, functional genomic studies of this parasite lagged behind those performed with T. brucei, for which an RNAi system was developed long ago (32, 33). Gene deletion from *Leishmania* was, however, mostly performed by homologous recombination (34–36). Establishment of the CRISPR-Cas9 system for *Leishmania* (16) now makes it easier to examine the effect of introducing null mutations of specific proteins, as well as to test the effects of heterologous deletions of only a single allele, especially when essential proteins are involved.

Here, we report the generation of a LeishIF4E1^–/–^ null mutant in which both LeishIF4E1 alleles were deleted by the CRISPR-Cas9 technology. The complete deletion of both alleles was verified by different methodologies, including PCR analysis of the genomic DNA, Western blot analysis of cell extracts using antibodies against LeishIF4E1, and mass spectrometry analysis of total protein extracts. LeishIF4E1 appears to be nonessential for parasite viability, but it did have a profound inhibitory effect on parasite metabolism, global translation, growth, morphology, and infectivity. The null mutant cells already had a rounded shape and possessed a very short flagellum under promastigote growth conditions. When exposed to elevated temperatures and acidic pH, the cells became smaller and adapted a morphology that resembled that of axenic amastigotes, although other features were not tested. However, when these cells returned to conditions specific for promastigote growth, the LeishIF4E1^–/–^ cells remained small and rounded for 3 days and returned to their original defective shape only after 4 days, when the cells developed a very short flagellum. For comparison, all the control cells resumed their elongated shape with a long flagellum after 24 h. These results were in agreement with the changes observed in the analysis of the total proteome content of the LeishIF4E1^–/–^ mutant compared to that of wild-type cells. The absence of LeishIF4E1 impaired cell morphology by downregulating the expression of paraflagellar-rod proteins, including its core components (PFR1 and PFR2), other proteins associated with the paraflagellar rod, and cytoskeletal components. The link between the severe effects of LeishIF4E1 deletion on parasite morphology was strengthened by complementing the expression of LeishIF4E1 in the add-back line. Cell morphology and growth rates resumed in the add-back cells, indicating that morphology impairment occurred due to the elimination of LeishIF4E1 from the parasite genome. The possibility that the reduction in the metabolic state of the cells could be the reason for the observed changes cannot be excluded, although the absence of LeishIF4E1 targeted very specific protein groups rather than having a general effect.

The LeishIF4E1^–/–^ null mutant parasites showed a reduced ability to enter and infect cultured macrophages, compared to that of control lines. This was observed by 1 h postinfection and following 24 h as well. Infectivity was measured both by counting the number of infected cells and by counting the number of parasites per infected macrophage. The parasites that entered the cells were viable, as their average number increased following 24 h of incubation. The impaired infectivity of the LeishIF4E1^–/–^ cells may be related to the lack of a normal flagellum and also to the reduced expression of surface proteins that are essential for survival in the host cells. Both GP63 and GP46/PSA have been reported to contribute to parasite virulence, and both are downregulated in LeishIF4E1^–/–^ cells. GP63 is a surface protease that cleaves the complement factor C3b into iC3b, preventing complement-mediated lysis of the parasites (19). Upon entry into the macrophage, GP63 was shown to be involved in arresting the transcription (20) and translation (21) of the host macrophage by cleavage of specific factors involved in these processes. GP46/PSA, a known surface antigen, was also reported to promote parasite virulence, as it is involved in resistance to lysis by complement (22). The impaired infectivity may also be related to the strong reduction in the expression of cysteine peptidase (LmxM.20.1190) and the metallopeptidase LmxM.20.1191 (Table S1). Cysteine peptidases are also associated with virulence in *Leishmania* (37).

Involvement of the flagellum in parasite infectivity is well documented (38–40). The inability to synthesize a typical flagellum in the LeishIF4E1^–/–^ mutant is reflected also in the reduced expression of paraflagellar-rod proteins. The downregulated proteins included the core proteins PFR1 and PFR2, in addition to Ca^2+^-sensing and calmodulin-binding proteins (18, 41). Overall, the reduced infectivity of LeishIF4E1^–/–^ cells can be attributed to the combined effects of impaired flagellar growth and reduced expression of various factors involved in virulence, such as surface proteins and cysteine peptidases. However, we do not exclude the possibility of other factors being involved.

The LeishIF4E1 deletion had an inhibitory effect on cell growth, which was observed mainly at the early and mid-log phases, as shown in Fig. 3D and E. We propose that this may be related to differences in the cap-binding activities of LeishIF4E1 along the growth curve, which were highest in the mid-log phase and reduced in stationary-phase cells. Mid-log-phase cells were also more active in their translation activity (N. Tupperwar, unpublished data). Thus, the effect of LeishIF4E1 elimination is more apparent in mid-log-phase cells and less in the later stages of growth. A growth defect caused by the elimination of the kinetoplastid IF4E1 was also observed in T. brucei bloodstream-form parasites in which expression of TbIF4E1 was eliminated by RNAi (42). Differentiation to the stumpy form of these T. brucei cells was normal, but they appeared to be unable to grow as procyclic forms. Overall, it appears that the impairment in promastigote growth of parasites that do not express the LeishIF4E1 ortholog is shared between *Leishmania* and *Trypanosoma.* Tethering experiments performed with TbIF4E-1 of T. brucei did not show any inhibitory effect of TbIF4E1 on the tethered reporter protein when it was tethered alone. An inhibitory effect on reporter expression was observed only when it was tethered with its interacting partner, Tb4E-IP. The latter served as a translation repressor, with or without TbIF4E1 (42). Our experiments with *Leishmania* do not support the idea that LeishIF4E1 is a translation repressor. On the contrary, its deletion led to a decrease in the global translation activity of the cells, while the recovery of LeishIF4E1 expression resumed translation rates.

We conclude that LeishIF4E1 deletion does not affect the viability of the parasites, but several promastigote features are highly compromised in its absence. Our recent publication on the generation of a LeishIF4E-3^+/–^ heterologous deletion mutant emphasizes the differences between the two proteins. While LeishIF4E1 is nonessential for parasite viability and both alleles could be eliminated, similar attempts to delete the two LeishIF4E3 alleles were unsuccessful. However, eliminating even a single gene copy of LeishIF4E3 resulted in parasites with impaired growth and infectivity, along with altered morphology and restricted growth of their flagellum (43). The involvement of both LeishIF4Es in the regulation of cell morphology and flagellar growth suggests that different LeishIF4Es may have overlapping cellular functions, as deletion of both cap-binding paralogs leads to comparable phenotypes.

## MATERIALS AND METHODS

### Cells.

Leishmania mexicana M379 cells were cultured at 25°C in medium 199 (M199; pH 7.4) supplemented with 10% fetal calf serum (FCS; Biological Industries), 5 μg/ml hemin, 0.1 mM adenine, 40 mM HEPES, 4 mM l-glutamine, 100 U/ml penicillin, and 100 μg/ml streptomycin.

RAW 264.7 macrophages were grown at 37°C in Dulbecco’s modified Eagle’s medium (DMEM) supplemented with 10% FCS, 4 mM l-glutamine, 0.1 mM adenine, 40 mM HEPES, pH 7.4, 100 U/ml penicillin, and 100 μg/ml streptomycin in an atmosphere of 5% CO_2_.

### Monitoring morphological changes in cells exposed to conditions known to induce axenic differentiation and following their reversal to conditions typical of promastigote growth.

Promastigotes from all L. mexicana cell lines were seeded at a concentration of 5 × 10^5^/ml and grown for 3 days to reach their late log phase of growth (the concentrations reached were ∼3.6 × 10^7^ cells/ml for the wild type, ∼3.5 × 10^7^ cells/ml for Cas9/T7, ∼1.8 × 10^7^ cells/ml for the LeishIF4E1^–/–^ mutant, and ∼3.5 × 10^7^ cells/ml for add-back cells). The cells were washed twice with phosphate-buffered saline (PBS) and resuspended in M199 adjusted to pH 5.5 (by the addition of 0.5 M succinic acid) and supplemented with 25% FCS, 5 μg/ml hemin, 0.1 mM adenine, 40 mM HEPES, pH 5.5, 4 mM l-glutamine, 100 U/ml penicillin, and 100 μg/ml streptomycin. Cells were allowed to grow and differentiate at 33°C for 4 days under gentle-shaking conditions.

To allow the transformation of L. mexicana axenic amastigotes back to promastigotes, the cells were washed twice with PBS, resuspended in the medium used for promastigote growth (M199, pH 7.4), and transferred to 25°C.

### CRISPR-Cas9-mediated deletion of LeishIF4E1.

Plasmids developed for the CRISPR system in *Leishmania* were obtained from Eva Gluenz (University of Oxford, UK) (16). The pTB007 plasmid contained the genes encoding the Streptococcus pyogenes CRISPR-associated protein 9 endonuclease and the T7 RNA polymerase gene (Cas9/T7), along with the hygromycin resistance gene. pTB007 was transfected into L. mexicana promastigotes, and transgenic cells stably expressing Cas9 and the T7 RNA polymerase were selected for hygromycin resistance (200 μg/ml).

### Generation of the LeishIF4E1 deletion mutant by CRISPR-Cas9.

To generate LeishIF4E1 null mutants, we used three PCR-amplified products: the two 5′ and 3′ sgRNAs designed to create double-strand breaks upstream and downstream of the LeishIF4E1 coding region and the LeishIF4E1 repair cassette fragment containing the G418 resistance marker. The three PCR products were transfected into mid-log-phase transgenic cells expressing Cas9 and T7 RNA polymerase, and cells were further selected for resistance to 200 μg/ml G418 (44). Thus, the LeishIF4E1^–/–^ line may be polyclonal.

The sgRNA sequences that were used to delete the LeishIF4E1 gene were obtained from http://leishgedit.net/ (45). The sgRNAs contained the highest-scoring 20-nucleotide sequence within 105 bp upstream or downstream of the target gene. The sequences of the sgRNAs were subjected to a BLAST search against the L. mexicana genome in TriTrypDB to verify the specificities of the sgRNAs for LeishIF4E1 (E values = 0.001 and 8e−5). We also ran a BLAST analysis with the drug resistance repair cassette that contained the sequence with homology to the UTR of LeishIF4E1 to specifically target the insertion of the selection marker. The repair cassette showed an E value of 5e−9, suggesting a very high specificity of the system. The sgRNA target sequences and the homology arms on the repair cassette fully matched the target sequence of LeishIF4E1.

### PCR amplification of sgRNA templates.

DNA fragments encoding LeishIF4E1-specific 5′ and 3′ guide RNAs for cleavage upstream and downstream of the LeishIF4E1 target gene were generated. The template for this PCR consisted of two fragments; one contained the common sgRNA scaffold fragment (5′-AAAGCACCGACTCGGTGCCACTTTTTCAAGTTGATAACGGACTAGCCTTATTTTAACTTGCTATTTCTAGCTCTAAAAC-3′), and the other contained the T7 RNA polymerase promoter (lowercase letters at the beginning of the sequence below) fused to the gRNA (5′ or 3′) targeting LeishIF4E1 (capital letters below) and a short sequence overlapping the scaffold fragment (lowercase letters at the end of the sequence below). The two individual template fragments for targeting a double-strand break at the 5′ end of LeishIF4E1 was (5′-gaaattaatacgactcactataggCTCTTCTTCGTTTCGCGCCAgttttagagctagaaatagc-3′), and the template fragment targeting a double-strand break at the 3′ end was (5′-gaaattaatacgactcactataggGTGTGCATATCATCTTGCTGgttttagagctagaaatagc-3′). Each of these two fragments (1 μM each) was annealed to the partially overlapping scaffold fragment and further amplified with two small primers (2 μM each) derived from the T7 promoter (G00F, 5′-TTAATACGACTCACTATAGG-3′) and the common scaffold fragment (G00R, 5′-GCACCGACTCGGTGCCACTT-3′). The reaction mixture consisted of deoxynucleoside triphosphates (dNTPs) (0.2 mM) and HiFi polymerase (1 unit of Phusion; NEB) in buffer suitable for templates rich in GC (guanosine and cytosine) with MgCl_2_ (NEB) in a total volume of 50 μl. PCR conditions included initial denaturation at 98°C for 2 min, which was followed by 35 cycles of 98°C for 10 s, annealing at 60°C for 30 s, and an extension at 72°C for 15 s. All PCR products were gel purified and heated at 94°C for 5 min before transfection.

### PCR amplification of the LeishIF4E1 replacement fragment.

A DNA fragment designed to repair the double-strand breaks surrounding the LeishIF4E1 target gene was amplified by PCR. The LeishIF4E1-specific primers were derived from the 5′ and 3′ endogenous UTR sequences upstream and downstream of the LeishIF4E1 gene and the sequences from the antibiotic repair cassette, based on sequences in the LeishGEdit database (http://www.leishgedit.net/Home.html). The primers were (5′-CTAGATCATCGCCTTACGCACCCCCCTCCCgtataatgcagacctgctgc-3′ [forward]) and 5′-CACAACACGTAGACAAGCAAACATCAACCAccaatttgagagacctgtgc-3′ [reverse]). Capital letters represent the UTR sequences of LeishIF4E1, and lowercase letters represent the region on the pT plasmid that flanks the UTR adjacent to the antibiotic resistance gene. The PCR for generating the fragment used for repair of the double-strand breaks on both sides of the gene targeted for deletion was performed using the pTNeo plasmid as a template. The resulting fragments enable the integration of the drug resistance marker by homologous recombination at the target site. The reaction mixture consists of 2 μM each primer, dNTPs (0.2 mM), the template (pTNeo at 30 ng), 3% (vol/vol) dimethyl sulfoxide (DMSO), and HiFi polymerase (1 unit of Phusion; NEB) in GC buffer (containing MgCl_2_ to a final concentration of 1.5 mM) in a total volume of 50 μl. PCR conditions included initial denaturation at 98°C for 4 min, which was followed by 40 cycles of 98°C for 30 s, annealing at 65°C for 30 s, and an extension at 72°C for 2 min 15 s. The final extension was performed for 7 min at 72°C. All PCR products were gel purified and heated at 94°C for 5 min before transfection.

### Diagnostic PCR to confirm the deletion of LeishIF4E1.

Genomic DNA from the drug-resistant cells was isolated 14 days posttransfection using a DNeasy blood and tissue kit (Qiagen) and analyzed for the presence of the LeishIF4E1 gene, using specific primers derived from the open reading frame (ORF) of LeishIF4E1. The primers used were LeishIF4E1 forward (5′-GGATCCATGTCATCTCCATCTTCAG-3′) and LeishIF4E1 reverse (5′-TCTAGAAGACGCCTCGCCGTGCTT-3′). A parallel reaction was performed to look for the presence of the G418 resistance gene, with primers derived from its ORF: G418 F (5′-GCCCGGTTCTTTTTGTCAAGAC-3′) and G418 R (5′-GTCACGACGAGATCATCATCGCCG-3′). Genomic DNA from Cas9/T7 L. mexicana cells was used as a positive control for the presence of the LeishIF4E1 gene. The reaction mixture consisted of 2 μM each primer, genomic DNA (gDNA) (100 ng), dNTPs (0.2 mM), and HiFi polymerase (1 unit of Phusion; NEB) in GC buffer with MgCl_2_ (NEB) in a total volume of 50 μl. PCR conditions included initial denaturation at 98°C for 4 min, which was followed by 35 cycles of 98°C for 30 s, annealing at 60°C for 30 s, and an extension at 72°C for 2 min 15 s. A final extension was done for 7 min at 72°C. PCR products were separated over 1% agarose gels.

### Generation of LeishIF4E1 add-back parasites.

The transgenic LeishIF4E1^–/–^ deletion mutant cells were transfected with an episomal transfection vector that promoted the recovery of LeishIF4E1 expression. The plasmid was derived from pTPuro, which confers resistance to puromycin. The added-back LeishIF4E1 gene from L. mexicana was tagged with the streptavidin-binding peptide (SBP; ∼4 kDa), which enabled its further identification in the transgenic parasites by antibodies against the SBP tag (14). Tagged LeishIF4E1 was cloned between two intergenic regions derived from the HSP83 (H) genomic cluster. Stably transfected cells were selected for resistance to puromycin. The pTPuro-LeishIF4E1 plasmid was generated as follows. The ORF of LeishIF4E1 from L. mexicana was amplified using the forward 5′-ggatccATGTCATCTCCATCTTCAG-3′ and reverse 5′-tctagaAGACGCCTCGCCGTGCT-3′ primers, with BamHI and XbaI sites introduced at the 5′ ends of these primers (lowercase letters). The BamHI/XbaI PCR product was cloned into the BamHI and XbaI sites of the pX-H-SBP-H expression cassette between two intergenic regions derived from the HSP83 genomic locus (46, 47). The fragment containing the SBP-tagged LeishIF4E1 ORF and the two flanking HSP83 intergenic regions was cleaved by HindIII, blunted, and cloned into the blunted SfoI site of pT-Puro (16). The resulting pTPuro-LeishIF4E1-SBP expression vector was transfected into the LeishIF4E1^–/–^ deletion mutant, and cells were selected for their resistance to puromycin (200 μg/ml).

### Growth analysis.

L. mexicana M379 wild-type and Cas9/T7-expressing cells, along with the LeishIF4E-1^–/–^ deletion mutant and the LeishIF4E-1 add-back cells, were cultured as promastigotes at 25°C in M199 containing all supplements (see above). Cells were seeded at a concentration of 5 × 10^5^ cells/ml, and the cells were counted daily for five consecutive days. The curves were obtained from three independent repeats.

### Western blot analysis.

Cells at their mid-log phase of growth (10 ml) were harvested and washed twice with PBS and once with post-ribosomal supernatant (PRS) buffer (35 mM HEPES, pH 7.5, 100 mM KCl, 10 mM MgCl_2_, 1 mM dithiothreitol [DTT]). The cell pellet was resuspended in PRS+ (300 μl), which was supplemented with a 2× cocktail of protease inhibitors (Sigma) and 4 mM iodoacetamide (Sigma), along with the following phosphatase inhibitors: 25 mM sodium fluoride, 55 mM β-glycerophosphate, and 5 mM sodium orthovanadate. Cells were lysed by the addition of 65 μl of 5× Laemmli sample buffer and heated at 95°C for 5 min. Cell extracts (40 μl) were resolved by 10% SDS-PAGE, blotted, and further probed using specific primary and secondary antibodies.

Antibodies against LeishIF4E1 (rabbit polyclonal, 1:2,000) and against the SBP tag (Millipore; monoclonal, 1:10,000) were used to detect the endogenous and tagged LeishIF4E1 proteins, respectively. These were further detected by specific peroxidase-labeled secondary antibodies against rabbit (KPL; 1:10,000 for LeishIF4E1) and mouse (KPL; 1:10,000 for SBP).

### Translation assay.

Global translation was monitored using the SUnSET (surface sensing of translation) assay. This assay is based on the incorporation of puromycin, a tRNA analog, into the A site of translating ribosomes (48). Puromycin (1 μg/ml; Sigma) was added to cells for 30 min, which were then washed twice with PBS and once with PRS+. Cell pellets were resuspended in 300 μl of PRS+ buffer, denatured in Laemmli sample buffer, and boiled for 5 min. Cells treated with cycloheximide (100 μg/ml) prior to the addition of puromycin served as a negative control. Samples were resolved by 10% SDS-PAGE. The gels were blotted and subjected to Western blot analysis using monoclonal mouse antipuromycin antibodies (DSHB; 1:1,000) and secondary peroxidase-labeled anti-mouse antibodies (KPL; 1:10,000).

### XTT assay for measuring cell metabolism.

The metabolic activities of the different cells were estimated using a cell proliferation assay kit based on 2,3-bis [2-methoxy-4-nitro-5-sulfophenyl]-2 H-tetrazolium-5-carboxyanilide inner salt (XTT; Biological Industries, Israel). XTT is reduced by mitochondrial dehydrogenases by metabolically active cells, resulting in an orange formazan compound. L. mexicana WT and LeishIF4E1^–/–^ deletion mutant cells were cultivated in 96-well plates in phenol red-free M199. Reaction solution containing an activation solution and XTT reagent was added to each well, and the plate was incubated at 26°C for 6 h. Absorbance at 450 nm, with a reference at 630 nm, was measured with an enzyme-linked immunosorbent assay (ELISA) reader.

### Phase-contrast microscopy of *Leishmania* promastigotes.

Cells from different lines in their late-log phase of growth were harvested, washed, fixed in 2% paraformaldehyde in PBS, and mounted onto glass slides. Phase-contrast microscope images were captured at a ×100 magnification with a Zeiss Axiovert 200M microscope equipped with an AxioCam HRm charge-coupled device (CCD) camera.

### Flow cytometry analysis of *Leishmania*.

Cell viability was verified by incubation of the cells with 20 μg/ml propidium iodide (PI) for 30 min. The stained cells were analyzed using the ImageStream X Mark II imaging flow cytometer (Millipore) with a 60/0.9× objective. Data from channels representing bright-field as well as fluorescence (PI) emission at 488 nm (to evaluate cell viability) were recorded for 20,000 cells for each analyzed sample. IDEAS software generated the quantitative measurements of the focused single and live cells for all four examined cell strains. Cell shape was quantified using circularity and elongatedness features applied on the bright-field image processed by an adaptive-erosion mask. Representative scatterplots are shown for focused single cells and for circularity (cell shape). Recorded emission of the PI in the gated population evaluated cell viability.

### Data analysis.

IDEAS software was used to generate the quantitative measurements of images recorded for the examined cell population. The focus quality of each cell was first determined by measuring the gradient root mean square (RMS) value. The cells representing high RMS values in the histogram were gated to select cells in focus. In the second step, single-cell populations were gated from the scatterplot of the aspect ratio over the area to exclude cell aggregates. Further, the intensity of PI staining was used to exclude dead cells. The remaining living, single cells in focus were subjected to image analysis to determine cell morphology. To obtain cell shape, a customized adaptive-erosion mask was used on the bright-field channel, with a coefficient of 78. We further customized this mask to exclude the flagellum from the cell shape analysis. Further, circularity and elongatedness features were measured. A predetermined threshold value of 4 was set to define circularity. Elongatedness values represent the ratio between cell length and width. Representative scatterplots are presented for focused single cells and for circularity. Cell viability was measured by recording the emission of PI in the gated population. All data shown are from a minimum of three biological replicates. A similar approach was taken for generating a template adapted for measuring the structural features of axenic amastigotes. Furthermore, due to the tendency of axenic amastigotes to aggregate, different cell populations were gated to obtain only single rounded cells (resembling axenic amastigotes). The gated area excluded cell aggregates, debris, and elongated promastigotes in a specific area of the scatterplot. This template was further used to quantify the single, round, amastigote-like cells in all the cell lines that were analyzed.

### *In vitro* macrophage infection assay.

L. mexicana LeishIF4E1^–/–^ deletion mutants and add-back cells and wild-type and transgenic parasites expressing Cas9/T7 polymerase were seeded at the concentration of 5 × 10^5^ cells/ml and allowed to grow for 5 days to reach their stationary growth phase. Parasites (WT, ∼5 × 10^7^/ml; Cas9/T7, ∼4.9 × 10^7^/ml; LeishIF4E1^–/–^ mutant, ∼4.4 × 10^7^/ml; add-back cells, ∼5.1 × 10^7^/ml) were washed with DMEM and labeled by incubation with 10 μM carboxyfluorescein succinimidyl ester (CFSE) in DMEM at 25°C for 10 min. The cells were then washed with DMEM, counted, and used to infect RAW 264.7 macrophages at a ratio of 10:1. The macrophages (5 × 10^5^) were preseeded a day in advance in chambered slides (Ibidi). The macrophages were incubated with the parasites for 1 h in 300 μl and then washed three times with PBS and once in DMEM to remove extracellular parasites. The infected macrophages were either fixed immediately for further analysis by confocal microscopy or further incubated for 24 h at 37°C in an atmosphere containing 5% CO_2_. The infected macrophages were then processed for confocal microscopy as described below. A single representative section of Z-projections (maximum intensity) produced by Image J software is presented in all the figures. With the cell-counting plug-in in Image J, the infectivity values were determined. We first counted the number of infected cells in a total of 200 macrophages and then counted the number of internalized parasites within the infected cells 1 or 24 h following infection. Statistics were generated using GraphPad Prism 5. We used the nonparametric Kruskal-Wallis test to determine significant differences in the infectivities and in the average numbers of parasites per infected macrophage.

### Confocal microscopy of *Leishmania* promastigotes.

Following 1 or 24 h of infection, the macrophages were washed with PBS, fixed in 2% paraformaldehyde for 30 min, washed once with PBS, and permeabilized with 0.1% Triton X-100 in PBS for 10 min. We stained nucleic acids with 4′,6-diamidino-2-phenylindole (DAPI; 1 μg/ml; Sigma), and finally, the cells were washed three times with PBS. The slides were observed using an inverted Zeiss LSM 880 Axio Observer Z1 confocal laser-scanning microscope with an Airyscan detector. Cells were visualized using a Zeiss Plan-Apochromat oil lens objective of 63× and a numerical aperture of 1.4. Z-stacked images were acquired with a digital zoom of 8× (1.8× for broad fields), using the Zen lite software (Carl Zeiss microscopy). Images were processed using the Image J software package. A single representative section of the compiled Z-projections produced by Image J software is presented in all the figures.

### Mass spectrometry analysis.

To characterize the proteomic differences between the LeishIF4E1^–/–^ deletion mutant and wild-type cells, we carried out mass spectrometry analysis of total cell lysates of LeishIF4E1^–/–^ mutant cells. Total cell lysates from mid-log-stage promastigotes (WT, ∼3.4 × 10^7^/ml; Cas9/T7 cells, ∼3 × 10^7^/ml; LeishIF4E1^–/–^ cells, ∼1 × 10^7^/ml; and add-back cells, ∼3.2 × 10^7^/ml) were resuspended in a buffer containing 100 mM Tris-HCl, pH 7.4, 10 mM DTT, 5% SDS, 2 mM iodoacetamide, and a cocktail of protease inhibitors. Cell lysates were precipitated using 10% trichloroacetic acid (TCA), and the pellets were washed with acetone. The mass spectrometric analysis was performed by the Smoler Proteomics Center at the Technion in Israel.

### (i) Mass spectrometry.

Proteins were reduced using 3 mM DTT (60°C for 30 min), followed by modification with 10 mM iodoacetamide in 100 mM ammonium bicarbonate for 30 min at room temperature. This was followed by overnight digestion in 10 mM ammonium bicarbonate in trypsin (Promega) at 37°C. Trypsin-digested peptides were desalted, dried, resuspended in 0.1% formic acid, and resolved by reverse-phase chromatography over a 30-min linear gradient with 5% to 35% acetonitrile and 0.1% formic acid in water, a 15-min gradient with 35% to 95% acetonitrile and 0.1% formic acid in water, and a 15-min gradient at 95% acetonitrile and 0.1% formic acid in water at a flow rate of 0.15 μl/min. MS was performed using a Q-Exactive Plus mass spectrometer (Thermo) in positive mode set to conduct a repetitively full MS scan followed by high-energy collision dissociation of the 10 dominant ions selected from the first MS scan. Mass tolerances of 10 ppm for precursor masses and 20 ppm for fragment ions were set.

### (ii) Statistical analysis for enriched proteins.

Raw mass spectrometric data were analyzed by the MaxQuant software, version 1.5.2.8 (49). The data were searched against the annotated L. mexicana proteins from TriTrypDB (50). Protein identification was set at less than a 1% false-discovery rate. The MaxQuant settings selected were a minimum of 1 razor per unique peptide for identification, a minimum peptide length of 6 amino acids, and a maximum of two miscleavages. For protein quantification, summed peptide intensities were used. Missing intensities from the analyses were replaced with values close to baseline only if the values were present in the corresponding analyzed sample. The log_2_ values of label-free quantification (LFQ) intensities (51) were compared between the three biological repeats of each group on the Perseus software platform (17), using a *t* test. The enrichment threshold was set to a log_2_ fold change of >1.6 and a *P* of <0.05. The annotated proteins were first categorized manually.

### (iii) Categorization of enriched proteins by the GO annotation via TriTrypDB.

Enriched proteins were classified by the GO annotation tool in TriTrypDB, based on molecular functions. The threshold for the calculated enrichment of proteins based on their GO terms was set at 2.5-fold, with a *P* of <0.05. This threshold eliminated most of the general groups that represented parental GO terms. GO terms for which only a single protein was annotated were filtered out as well. In some cases, GO terms that were included in other functional terms are not shown, leaving only the representative GO term.

### Statistical analysis.

Statistical analysis was performed using GraphPad Prism version 5. Each experiment was performed independently at least three times, and the individual values are presented as dots. For experiments with a higher number of repeats, results are expressed as means ± standard deviations (SD). Statistical significance was determined using the Wilcoxon paired *t* test for matched pairs or the Kruskal-Wallis test with Dunn’s multiple-comparison test for comparing three or more groups. Significant *P* values were marked as follows: *P* <0.05 (*), *P *< 0.01 (**), and *P *< 0.001 (***).
