# Supplementary figures and images for: LeishIF4E1 Deletion Affects the Promastigote Proteome, Morphology, and Infectivity
Source: mSphere. 2019 Nov 13;4(6):e00625-19. doi: 10.1128/mSphere.00625-19 (PMC6854042; doi:10.1128/mSphere.00625-19)

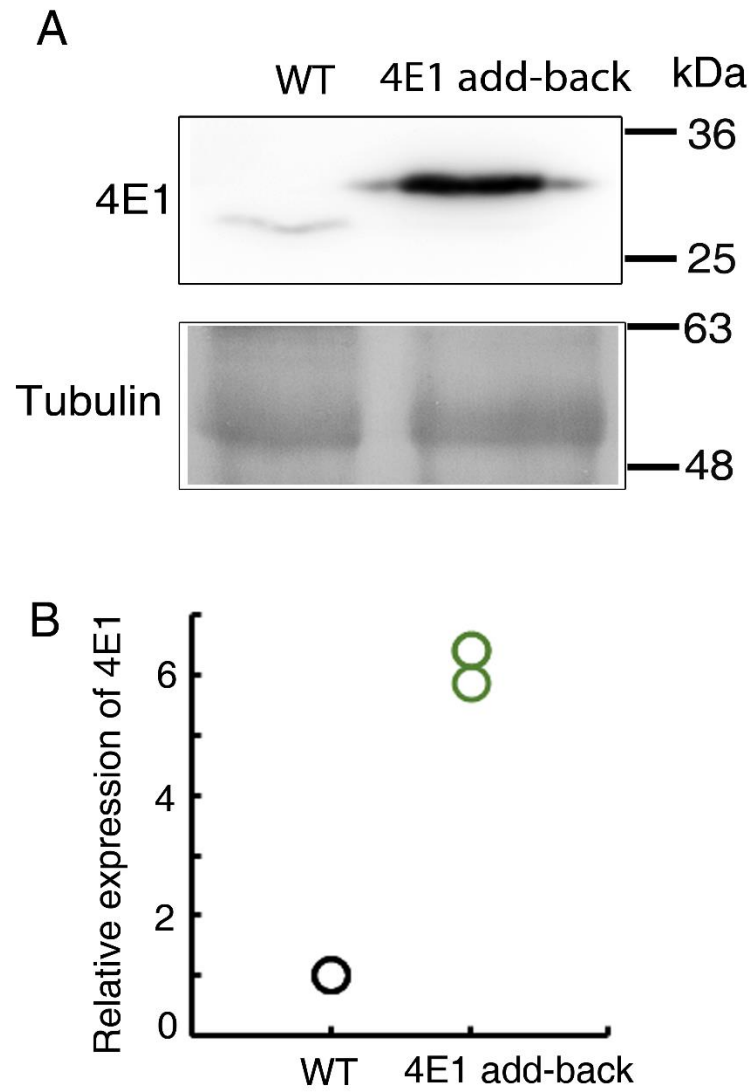

**Figure S1**

Supplement: FIG S1 [file mSphere.00625-19-sf001.pdf]

**A**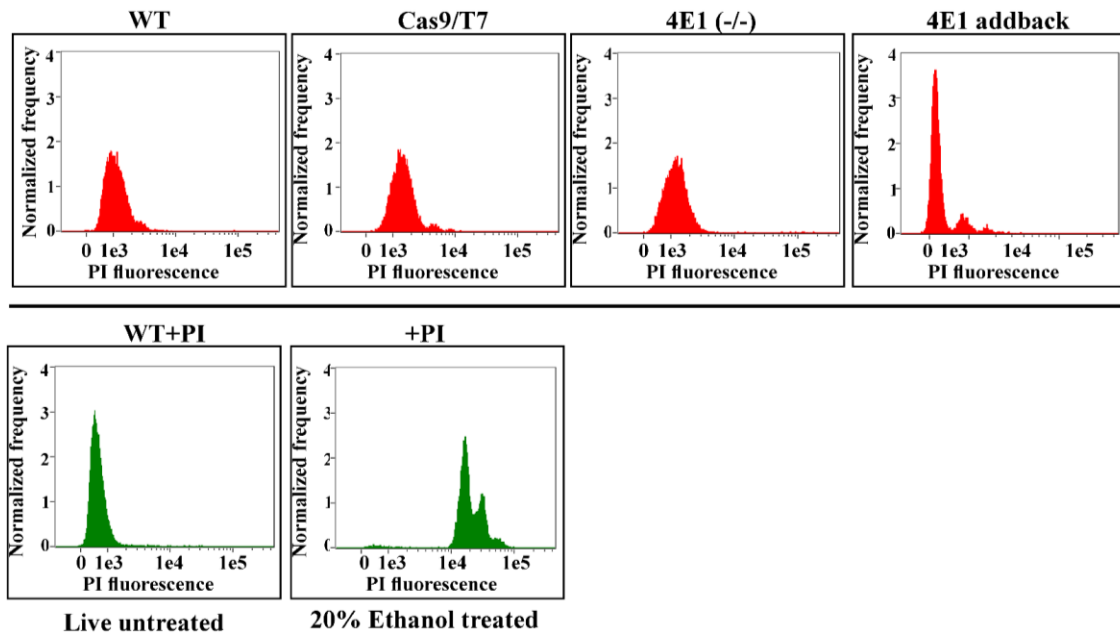**B**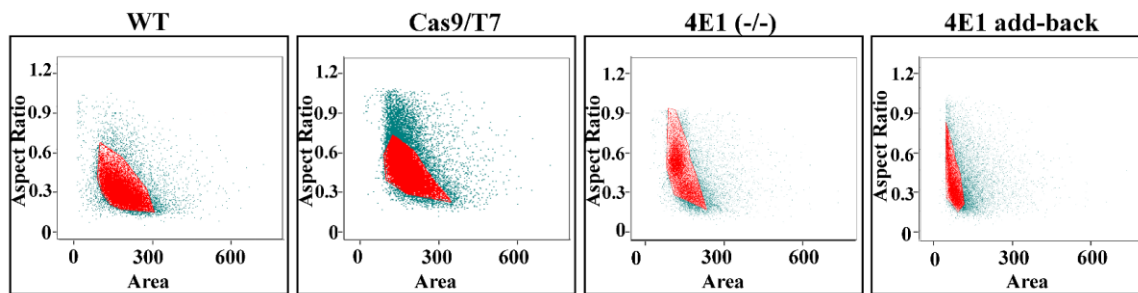**C**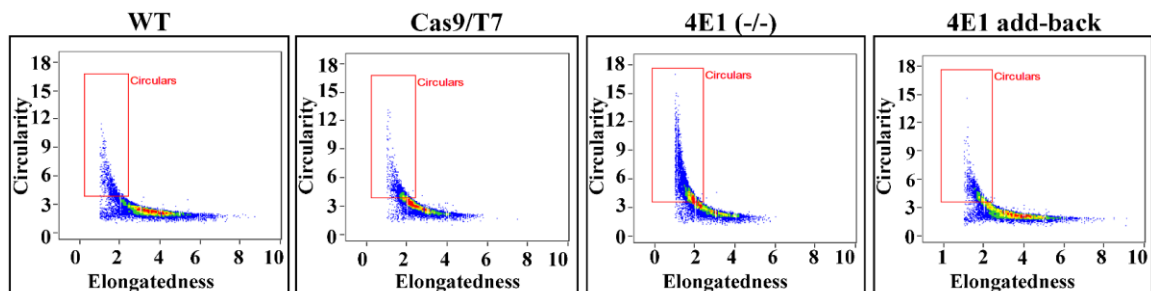**Figure S2**

Supplement: FIG S2 [file mSphere.00625-19-sf002.pdf]

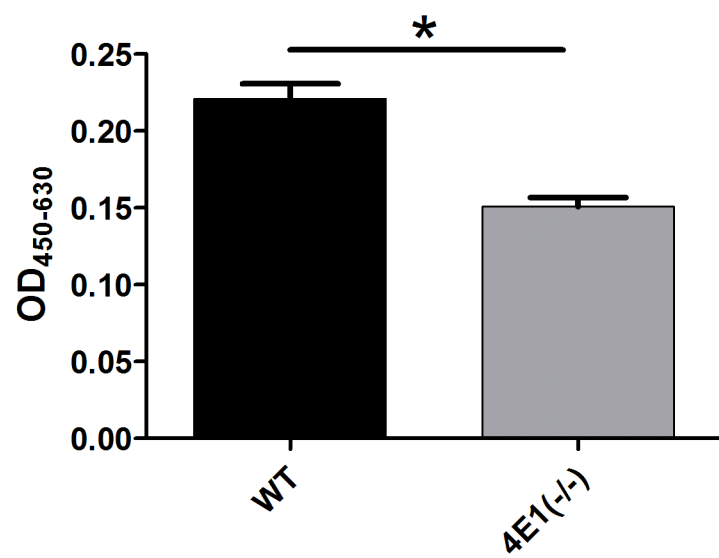

Figure S3

Supplement: FIG S3 [file mSphere.00625-19-sf003.pdf]

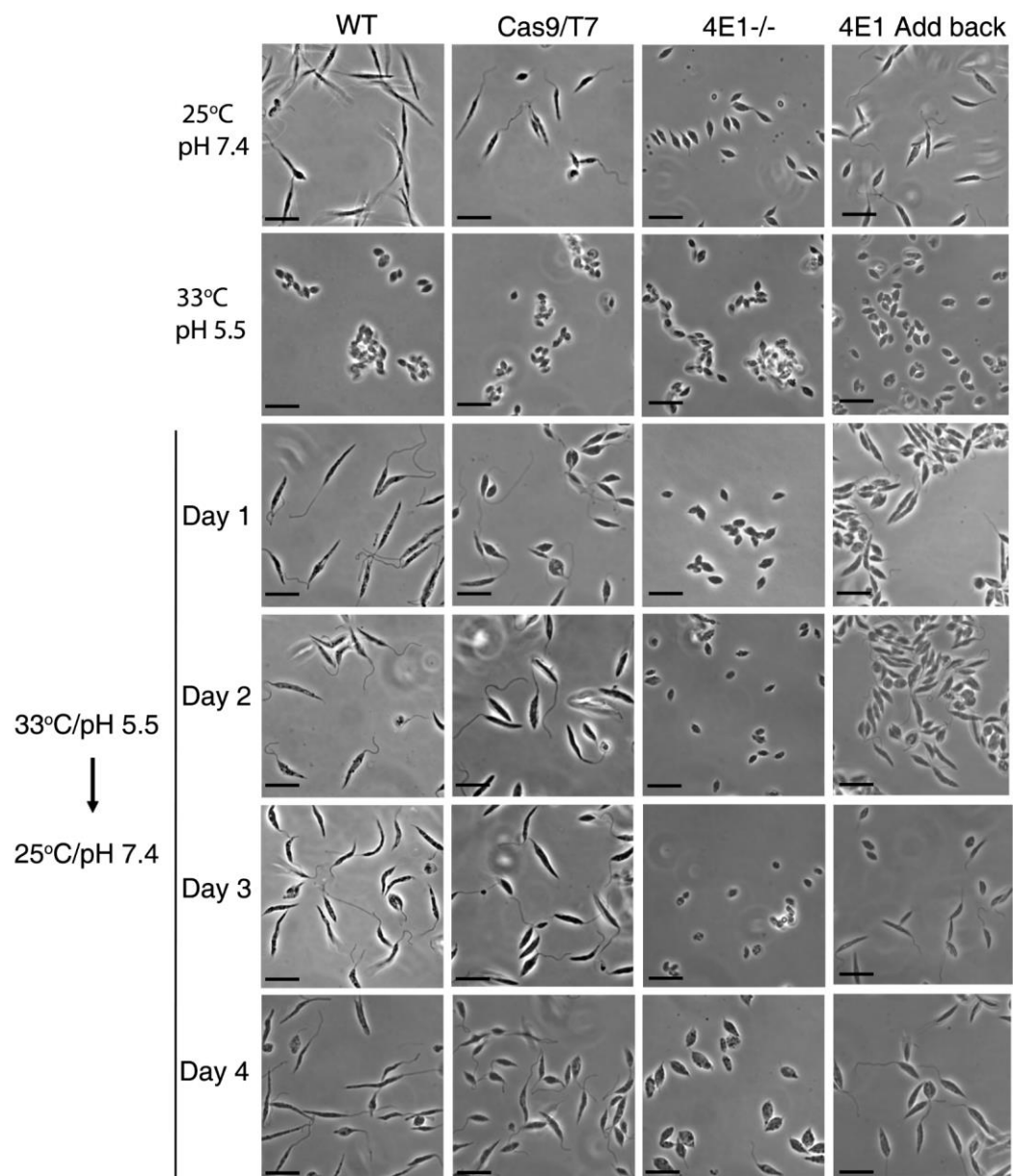

**Figure S4**

Supplement: FIG S4 [file mSphere.00625-19-sf004.pdf]

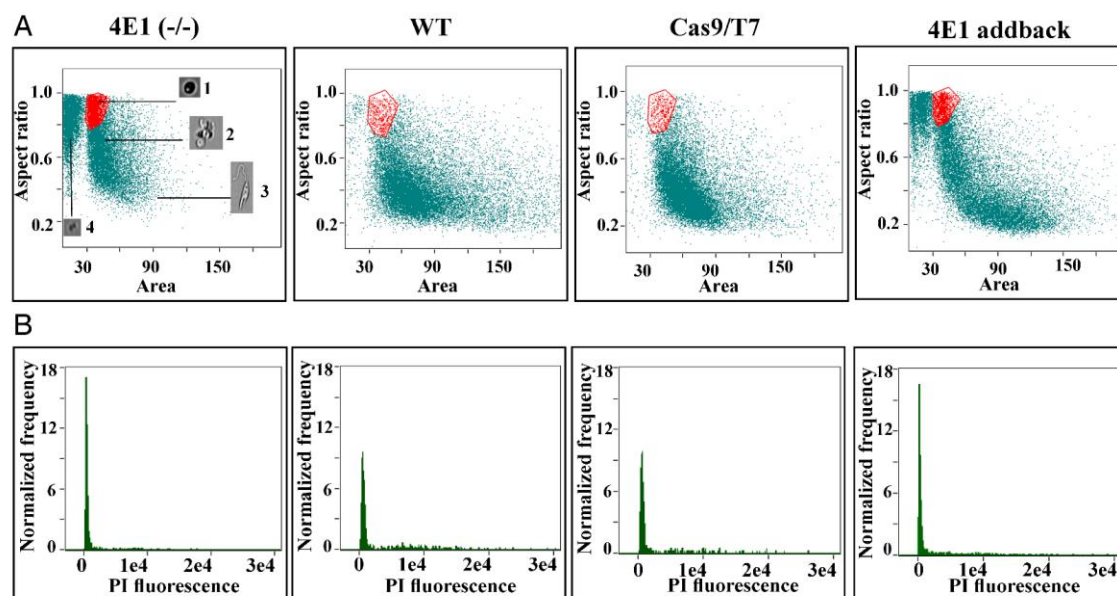

**Figure S5**

Supplement: FIG S5 [file mSphere.00625-19-sf005.pdf]

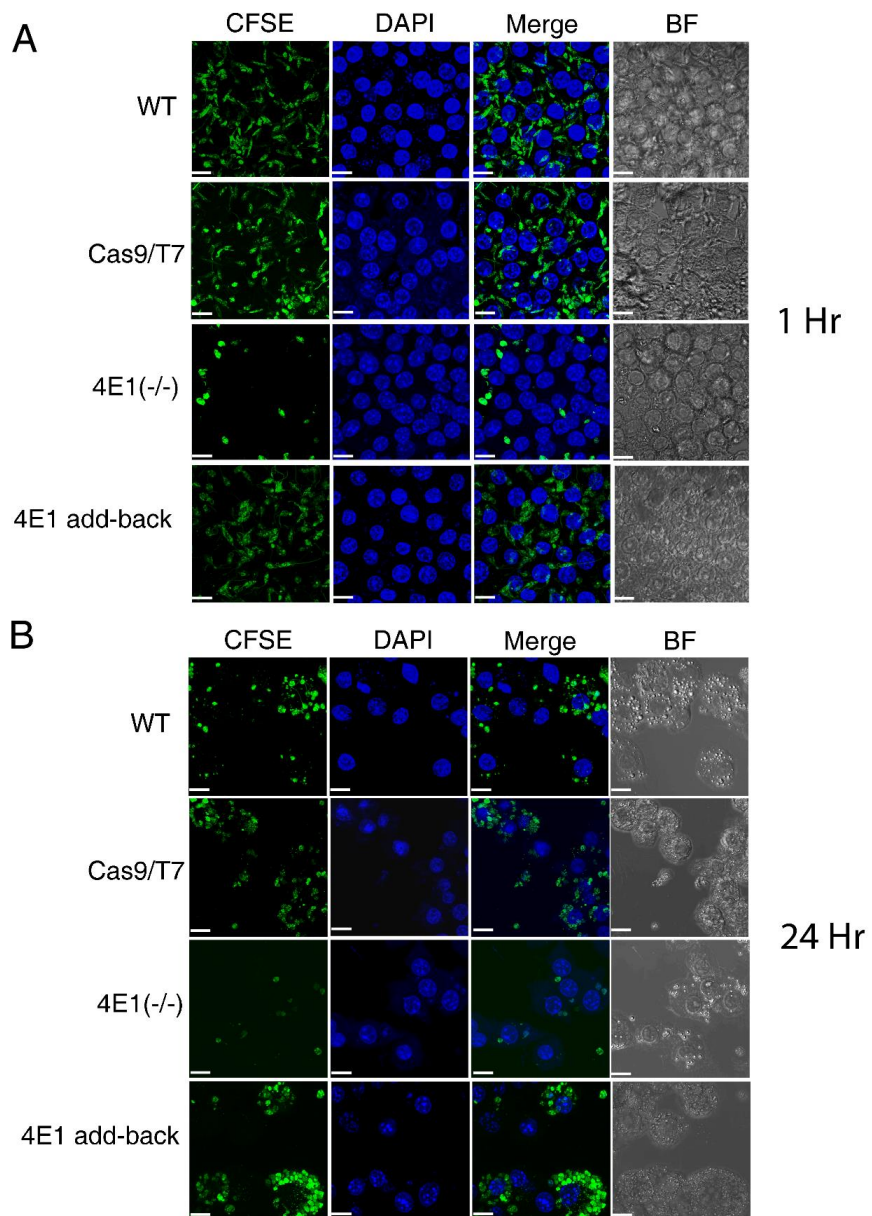

**Figure S6**

Supplement: FIG S6 [file mSphere.00625-19-sf006.pdf]

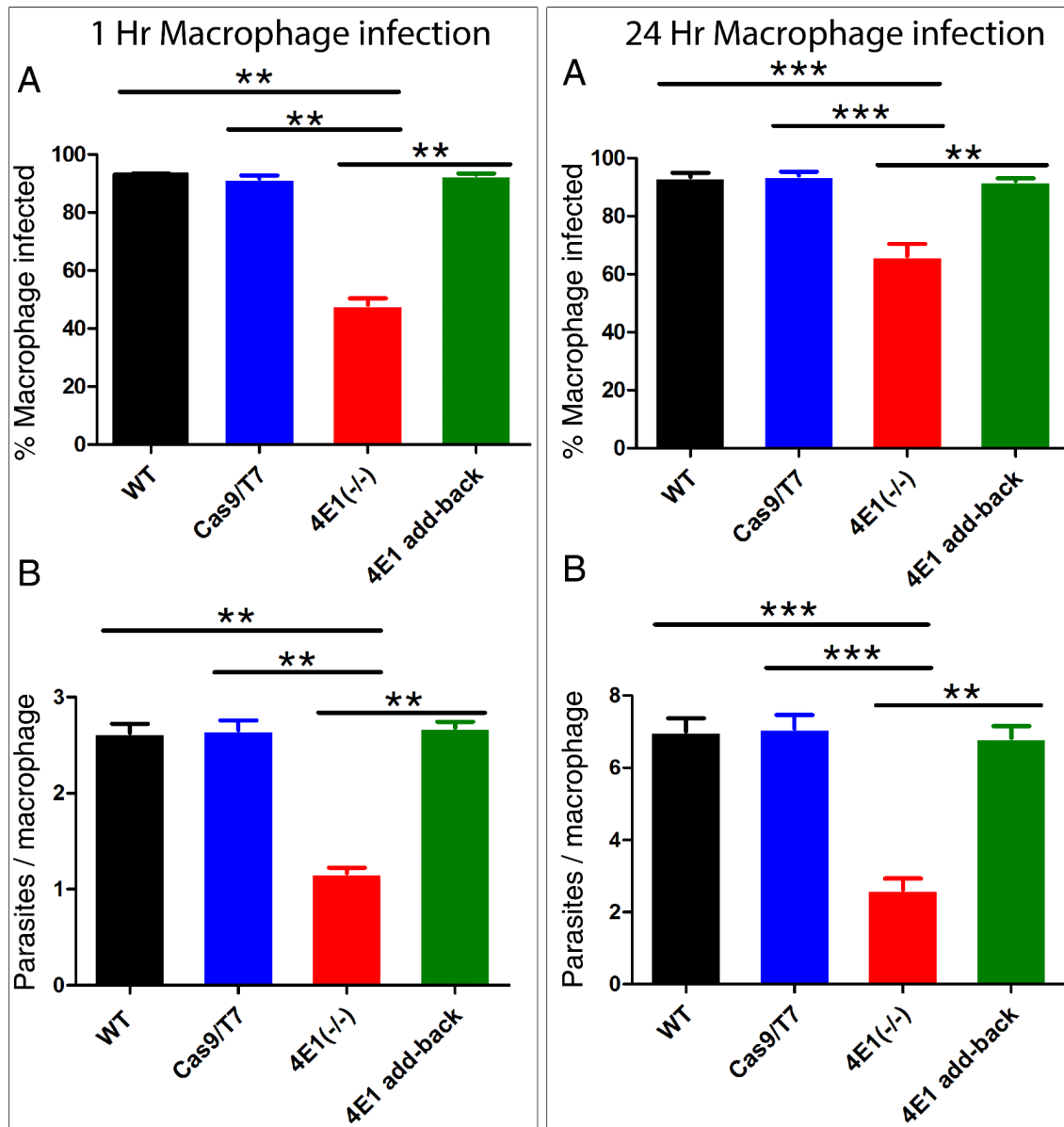

Figure S7

Supplement: FIG S7 [file mSphere.00625-19-sf007.pdf]

A

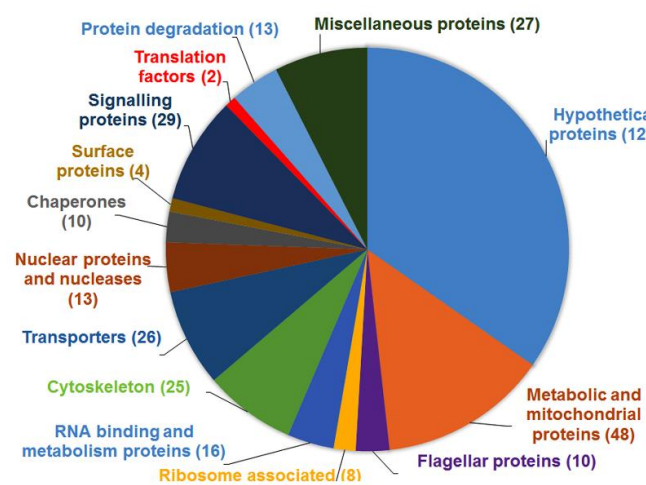

B

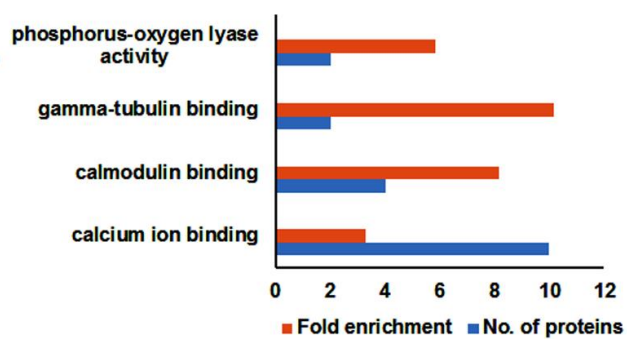

Figure S8

Supplement: FIG S8 [file mSphere.00625-19-sf008.pdf]
